# *Pup1* QTL Regulates Gene Expression Through Epigenetic Modification of DNA Under Phosphate Starvation Stress in Rice

**DOI:** 10.3389/fpls.2022.871890

**Published:** 2022-05-31

**Authors:** Suresh Kumar, Karishma Seem, Santosh Kumar, K. K. Vinod, Viswanathan Chinnusamy, Trilochan Mohapatra

**Affiliations:** ^1^Division of Biochemistry, ICAR-Indian Agricultural Research Institute, New Delhi, India; ^2^Decode Genomics Private Limited, New Delhi, India; ^3^Division of Genetics, ICAR-Indian Agricultural Research Institute, New Delhi, India; ^4^Division of Plant Physiology, ICAR-Indian Agricultural Research Institute, New Delhi, India; ^5^Indian Council of Agricultural Research, New Delhi, India

**Keywords:** DNA methylation, gene body methylation, phosphate starvation, gene expression, rice, RdDM pathway

## Abstract

Cytosine methylation, epigenetic DNA modification, is well known to regulate gene expression. Among the epigenetic modifications, 5-methylcytosine (5-mC) has been one of the extensively studied epigenetic changes responsible for regulating gene expression in animals and plants. Though a dramatic change in 5-mC content is observed at the genome level, the variation in gene expression is generally less than that it is expected. Only less is understood about the significance of 5-mC in gene regulation under P-starvation stress in plants. Using whole-genome bisulfite sequencing of a pair of rice [Pusa-44 and its near-isogenic line (NIL)-23 harboring *Pup1* QTL] genotypes, we could decipher the role of *Pup1* on DNA (de)methylation-mediated regulation of gene expression under P-starvation stress. We observed 13–15% of total cytosines to be methylated in the rice genome, which increased significantly under the stress. The number of differentially methylated regions (DMRs) for hypomethylation (6,068) was higher than those (5,279) for hypermethylated DMRs under the stress, particularly in root of NIL-23. Hypomethylation in CHH context caused upregulated expression of 489 genes in shoot and 382 genes in root of NIL-23 under the stress, wherein 387 genes in shoot and 240 genes in root were upregulated exclusively in NIL-23. Many of the genes for DNA methylation, a few for DNA demethylation, and RNA-directed DNA methylation were upregulated in root of NIL-23 under the stress. Methylation or demethylation of DNA in genic regions differentially affected gene expression. Correlation analysis for the distribution of DMRs and gene expression indicated the regulation of gene mainly through (de)methylation of promoter. Many of the P-responsive genes were hypomethylated or upregulated in roots of NIL-23 under the stress. Hypermethylation of gene body in CG, CHG, and CHH contexts caused up- or downregulated expression of transcription factors (TFs), P transporters, phosphoesterases, retrotransposon proteins, and other proteins. Our integrated transcriptome and methylome analyses revealed an important role of the *Pup1* QTL in epigenetic regulation of the genes for transporters, TFs, phosphatases, carbohydrate metabolism, hormone-signaling, and chromatin architecture or epigenetic modifications in P-starvation tolerance. This provides insights into the molecular function of *Pup1* in modulating gene expression through DNA (de)methylation, which might be useful in improving P-use efficiency or productivity of rice in P-deficient soil.

## Introduction

Phosphorus (P), being one of the most important macronutrients for living organisms, is an important component of nucleic acids, membrane lipids, necessary for the proper functioning of enzymes, NADPH, ATP, etc., essential for energy-mediated metabolic processes, and vital for plant growth and development (Secco et al., 2015; Kumar et al., 2021b). Plants preferentially absorb this nutrient in the form of H_2_PO$H_{2}PO_{4}{}^{-}$ or HPO$HPO_{4}{}^{\text{-2}}$ from the soil, but its availability to plants is often hindered due to low solubility, immobility, and inaccessibility of P in the soil. For nutrient management, many times, a large amount of P fertilizers are used as the basal application in the soil. Moreover, increased doses of P fertilizers are used aiming at enhanced crop yield that not only increases the input cost but also causes the accumulation of harmful elements in the soil and environmental pollution (Chu et al., 2020). Due to the limited availability of rock phosphate (the major source of P fertilizers), indiscriminate use of P fertilizers may not be economically and ecologically sustainable in long run. Therefore, it becomes critical to understand the molecular mechanisms or pathways involved in inorganic phosphate (Pi) homeostasis toward the development of plants having better Pi acquisition and use efficiency for sustainable yield and global food security. Some of the plants deploy a set of sophisticated mechanisms for efficient acquisition and utilization to maintain cellular Pi homeostasis even under limited Pi availability (Rouached et al., 2010; Chiou and Lin, 2011; Secco et al., 2015; Kumar et al., 2021b).

Gene regulation at transcriptional and post-transcriptional levels is the well-known mechanism to cope with abiotic stresses; however, the recent evidence suggests that epigenetic modifications also play the important roles in regulating gene expression in response to the environmental cues, and the epigenetic marks may serve to memorize the stress (Secco et al., 2015; Kumar, 2018; Zahraeifard et al., 2018). Such epigenetic marks include, but are not limited to, DNA methylation, histone modifications, and chromatin architecture (Kumar et al., 2018, 2021a; Zahraeifard et al., 2018). In plants, DNA methylation may occur in all three sequence contexts (CG, CHG, and CHH, where H = A, C, or T), and different pathways are involved in the establishment, maintenance, and modification of DNA methylation pattern in these contexts (Law and Jacobsen, 2010; Wang et al., 2016). The epigenome is dynamic and remodeled effectively by the environmental cue (Manzano et al., 2012; Foroozani et al., 2020) to manage genome plasticity.

P-deficiency is one of the common abiotic stresses that affect plant growth, development, and productivity in >70% of the arable land world over (Vance, 2001). Plants have evolved a range of biochemical and physiological adaptive measures to cope with P deficiency including modulation in root system architecture (RSA) involving cell elongation, root meristem activity, and increased formation of lateral roots or root hairs (Sánchez-Calderón et al., 2005). Alterations in metabolic pathways have also been reported to optimize Pi utilization and remobilization within the plant, as well as the release of metabolites and enzymes to scavenge Pi from external sources that are not readily available for plant uptake (Morcuende et al., 2007; Kumar et al., 2021b, 2022).

Global gene expression analyses indicate that adaptive strategies are required for transcriptional activation or repression of a set of phosphate starvation-responsive (PSR) genes (Misson et al., 2005; Morcuende et al., 2007; Hsieh et al., 2009; Secco et al., 2015; Kumar et al., 2021b). In addition to the other regulatory mechanisms, Pi homeostasis is also regulated by post-transcriptional (Chiou and Lin, 2011), post-translational (Park et al., 2014), and epigenetic modifications (Sahu et al., 2013; Secco et al., 2015; Yong-Villalobos et al., 2015). Genome-wide variation in DNA methylation in response to low-P stress was reported in different plant species (Secco et al., 2015; Yong-Villalobos et al., 2015; Chu et al., 2020; Tian et al., 2021). The level of DNA (de)methylation depends on DNA methylation and demethylation activities catalyzed by several DNA methylase and demethylases (Cao and Jacobsen, 2002; Penterman et al., 2007; Li et al., 2018). Methylation or demethylation of TEs was reported in Arabidopsis, rice, and maize under abiotic stress (Garg et al., 2015; Yong-Villalobos et al., 2015; Mager et al., 2018). Dynamic DNA demethylation, accompanied by reduced siRNAs biogenesis, within TEs was reported by Chu et al. (2020) in soybean under low-P stress which regulated the transcriptional activities of transposons and the proximal PSR genes.

Modulations in chromatin architecture through histone modification and deposition of histone variant (H2A.Z) have also been reported to be involved in the activation of genes associated with Pi deficiency (Smith et al., 2010; Foroozani et al., 2020). Thus, epigenetic modification or chromatin remodeling plays an important role in modulating transcriptional activation or repression of PSR genes. However, the extent and the epigenetic marks that modulate the Pi-starvation or deficiency responses remain largely unknown. Stress-induced changes in DNA methylation of repetitive sequences or transposons have often been coupled with transcriptional changes in neighboring genes (Dowen et al., 2012; Tian et al., 2021). Differential expressions of DNA methylation regulators (methyltransferase 1, domains rearranged methylase 1, and DRM 2) were reported to be impacted by P-starvation stress in Arabidopsis (Yong-Villalobos et al., 2015).

Under P-starvation or deficiency stress, different plants adopt different strategies at different developmental stages and in different tissues. Increased expression of the genes coding for high-affinity P transporters (e.g., PHT1;6, PHT1;10) in roots increases Pi uptake, increased secretion of acid phosphatases (e.g., purple acid phosphatase) from root helps to solubilize the fixed P, and over-expression of ribonucleases in shoot helps to remobilize the bound Pi at the advanced stage of plant growth. Moreover, the level of P-containing intermediates such as nucleotides, RNA, and phospholipids is reduced or minimized under P-starvation or deficiency. In addition, phospholipids are replaced with sulfo- and galactolipids as the genes involved in sulfo- and galactolipid biosynthesis [sulfolipid synthases (SQDs) and monogalactosyldiacylglycerol synthases (MGDs)] are upregulated under P-starvation or deficiency (Misson et al., 2005; Secco et al., 2013; Kumar et al., 2021b, 2022). The requirement of Pi increases with the increasing age or biomass of the plant, which requires additional strategies to maintain P homeostasis. Members of the SPX (SYG1/Pho81/XPR1) domain-containing protein family (SPX, PHO1, and NLA), being involved in Pi transport and signaling, have also been reported to be the key regulators of Pi homeostasis (Wang et al., 2009, 2014d; Rouached et al., 2010; Kant et al., 2011; Secco et al., 2012, 2013; Puga et al., 2014). Multicopper oxidases were reported to be involved in the maintenance of P homeostasis in rice under P-deficiency stress (Cao et al., 2016). Similarly, Bcl-2-associated anthogenes (BAGs) are known to be involved in stress responses and modulate programmed cell death by controlling the rate of transcription both in plants and in animals (Doukhanina et al., 2006; Bansal et al., 2022). In addition, studies suggest that P homeostasis is also regulated through post-transcriptional mechanisms involving non-coding RNAs such as miR827, miR399, and IPS1 (Franco-Zorrilla et al., 2007; Chiou and Lin, 2011) as well as post-translational modifications (Park et al., 2014). Moreover, the expression of the gene-encoding DNA methyltransferase has been proposed to be directly controlled by PHR1, the master regulator of PSR (Yong-Villalobos et al., 2015). Dynamic remodeling of methylation patterns by nutrient stress might provide novel insight into the epimarks which could be utilized for varietal improvement in crops for nutrient-deficient soils.

Here, we report that P-starvation in contrasting rice genotypes induces significant changes in global DNA methylation and that the loss of DNA methylation in specific contexts alters the expression of several genes involved in morphological and physiological responses to P-starvation stress. Our data suggest the role of *Pup1* QTL in modulating expression of several PSR genes through epigenetic modification (DNA methylation) in rice; however, the involvement in other epigenetic factors is also evident.

## Materials and Methods

### Plant Materials and Genomic DNA Isolation

To analyze the effects of P-starvation on epigenetic modulation [changes in DNA cytosine (de)methylation] of gene expression in shoot and root of contrasting rice genotypes (Pusa-44, P-deficiency-sensitive; NIL-23, P-deficiency-tolerant), plants were grown hydroponically (refer to Kumar et al., 2021b) in PusaRicH medium supplied with (control) or without inorganic phosphorus (Pi) till vegetative or tillering (45 days old) stage of plant growth in three replications. Shoot and root tissues from the stress treated (P-starved, 0 ppm Pi) and control (P-sufficient, 16 ppm Pi) plants were collected in three replications (each replication containing the tissues from three plants), snap-frozen in liquid nitrogen, and stored at −80° C until used for molecular analyses. Genomic DNA was isolated in three replications using Qiagen DNeasy Mini kit (Qiagen) following the manufacturer's instructions. The quality or quantity of genomic DNA was estimated using Qubit-IV Fluorimeter (Life Technologies), and the integrity of the DNA was checked by agarose gel (0.8%) electrophoresis.

### Preparation of Library for Whole-Genome Bisulfite Sequencing

To prepare whole-genome bisulfite sequencing (WGBS) library, the genomic DNA isolated from three replications was pooled and fragmented to an average size of 200–500 bp using sonication (Covaris S220, Massachusetts, USA). After end-repair, adenylation, and adapter ligation (to protect from bisulfite conversion), the DNA fragments were treated with bisulfite using the ZYMO EZ DNA Methylation-Gold Kit (ZYMO Research, Orange County, CA, USA). The target fragments were excised from a 2% agarose gel and purified using QIAquick Gel Extraction Kit (Qiagen), and libraries were prepared by Illumina TruSeq-methylation kit. Finally, the libraries were got sequenced on the Illumina HiSeq 4000 platform (Illumina, San Diego, USA) using 150 bp paired-end chemistry with sufficient (~25 × ) coverage. Raw sequence data were submitted to NCBI Sequence Read Archive (SRA) database under the BioProject ID PRJNA802863.

### Read Processing, Alignment, and Identification of 5-MC

The adaptor sequences and low-quality reads were removed from the raw reads using FastQC toolkit (Andrews, 2010). The resultant high-quality reads for each sample were mapped to the rice reference genome (Rice Genome Annotation Project database, http://rice.plantbiology.msu.edu MSU v7.0) using Bismark (v0.8) software under default parameters (Krueger and Andrews, 2011). The efficiency of bisulfite conversion was estimated by mapping the high-quality filtered reads on the rice chloroplast genome. More than 99.5% of the cytosine(s) in the chloroplast genome were converted to thymine(s) indicating very high efficiency of bisulfite conversion. Methylcytosines (5-mCs) in the genome were identified based on the significance *p*-value of 0.001 and sequencing depth using a customized Perl script. Methylation level was determined by estimating the percentage of reads giving methylation call at a particular cytosine site to all the reads in the sequencing data.

### Identification of DMRs

Differentially methylated regions (DMRs) for changes in 5-mC content under P-starvation stress compared to the control (P-sufficient) for each genotype were determined by using CGmap Tools and a customized Perl script within each 100-bp bin in the rice genome. The bins covered by five reads and containing at least three cytosine residues were considered for differential methylation analysis. Differentially methylated bins showing at least 20% methylation level difference with *Q*-value (<0.05) calculated using Fisher's exact test and showing 2-fold difference were considered as DMRs. The DMRs were annotated through gene territories using GeneDMRs of R package (Wang et al., 2021b).

### Gene Ontology Analysis

To perform Gene Ontology (GO) enrichment analysis for the differentially methylated genes, AgriGO v2 (http://bioinfo.cau.edu.cn/agriGO) and ShinyGO v0.75 software (http://bioinformatics.sdstate.edu/go) were used. The analysis identifies enriched GO terms by comparing a query list of gene identifiers and their corresponding GO terms, with a background population list from which the query list was derived. The background list of genes and GO annotations were extracted from the Rice Genome Annotation Project database.

### RNA Isolation, Library Preparation, and Data Analysis

To analyze the effects of P-starvation on gene expression in the contrasting rice genotypes, total RNAs were isolated in three replications using TRIzol method. The RNA samples were used for transcriptome library preparation through the steps for mRNA enrichment, RNA fragmentation, first- and second-strand cDNA synthesis, purification, sequencing adaptor ligation, and PCR amplification following the manufacturer's instructions for TruSeq RNA Sample Preparation Kit (Illumina). The libraries were got sequenced commercially on Illumina platform using 150 bp PE chemistry. Raw sequence data for the libraries were submitted to NCBI Sequence Read Archive database under the BioProject ID PRJNA667189. Reference-based mapping of transcriptome data was performed using the rice reference genome (TIGR v7) using Hisat2 and StringTie package. The differentially expressed genes (DEGs) were annotated with Gene Ontology terms and key pathways *via* functional classification and Kyoto Encyclopedia of Gene and Genomes (KEGG) pathway mapping, respectively.

### Integration of DNA Methylation and Gene Expression Data

The correlation between DNA methylation and gene expression was determined by plotting methylation density of genes expressed at different levels in each sample based on fragments per kilobase of transcript per million (FPKM) value. Methylation density in the promoter, gene body, and flanking regions was estimated for the sets of genes expressed at varying levels. The correlation between differential methylation (2-fold change with <0.05 *Q*-value) and differential gene expression (2-fold change with <0.05 *p*-value) under P-starvation stress compared to control conditions within the same genotype was analyzed by estimating methylation level differences in different sequence contexts (CG, CHG, and CHH) within the gene body and flanking regions.

### Validation of Differentially Expressed/ Methylated Genes by RT-qPCR

To confirm the effect of cytosine methylation on gene expression, eight randomly selected differentially expressed genes (DEGs) in roots of the contrasting rice genotypes were used for quantitative (RT-qPCR) expression analysis following the MIQE guidelines. The expression level of three genes involved in DNA (de)methylation and RNA-directed DNA methylation (RdDM) pathway was also validated by RT-qPCR. Total RNAs were isolated using TRIzol reagent, treated with DNase I to remove any contaminating DNA, and subjected to reverse transcription (RT) using superscript II (Invitrogen). RT-qPCR was performed in 10 μl reaction mix on QIAquant 96 5plex machine (Qiagen, Germany) with 2 × KAPA SYBR fast qPCR Mastermix (KAPA Biosystems) following the manufacturer's instructions using gene-specific primers. The thermal cycler was programmed for an initial denaturation at 95°C for 3 min, followed by 39 cycles each of 20 s denaturation at 94°C, 20 s annealing at 60°C, and 40 s extension at 72°C. Amplification data collection was set at the end of each extension step. The data were analyzed through melt-curve analysis to check the specificity of PCR amplification. A total of three biological and three technical replicates were included for analysis of the data. The relative gene expression was determined by the 2^−Δ*ΔCT*^ method following the procedure mentioned elsewhere (Singh et al., 2015). Actin and tubulin genes were used as the internal references. The primers used for RT-qPCR validation are shown in Supplementary Table S1.

## Results

### Change in Methylation Landscape in Response to P-Starvation Stress

**Figure 1:**
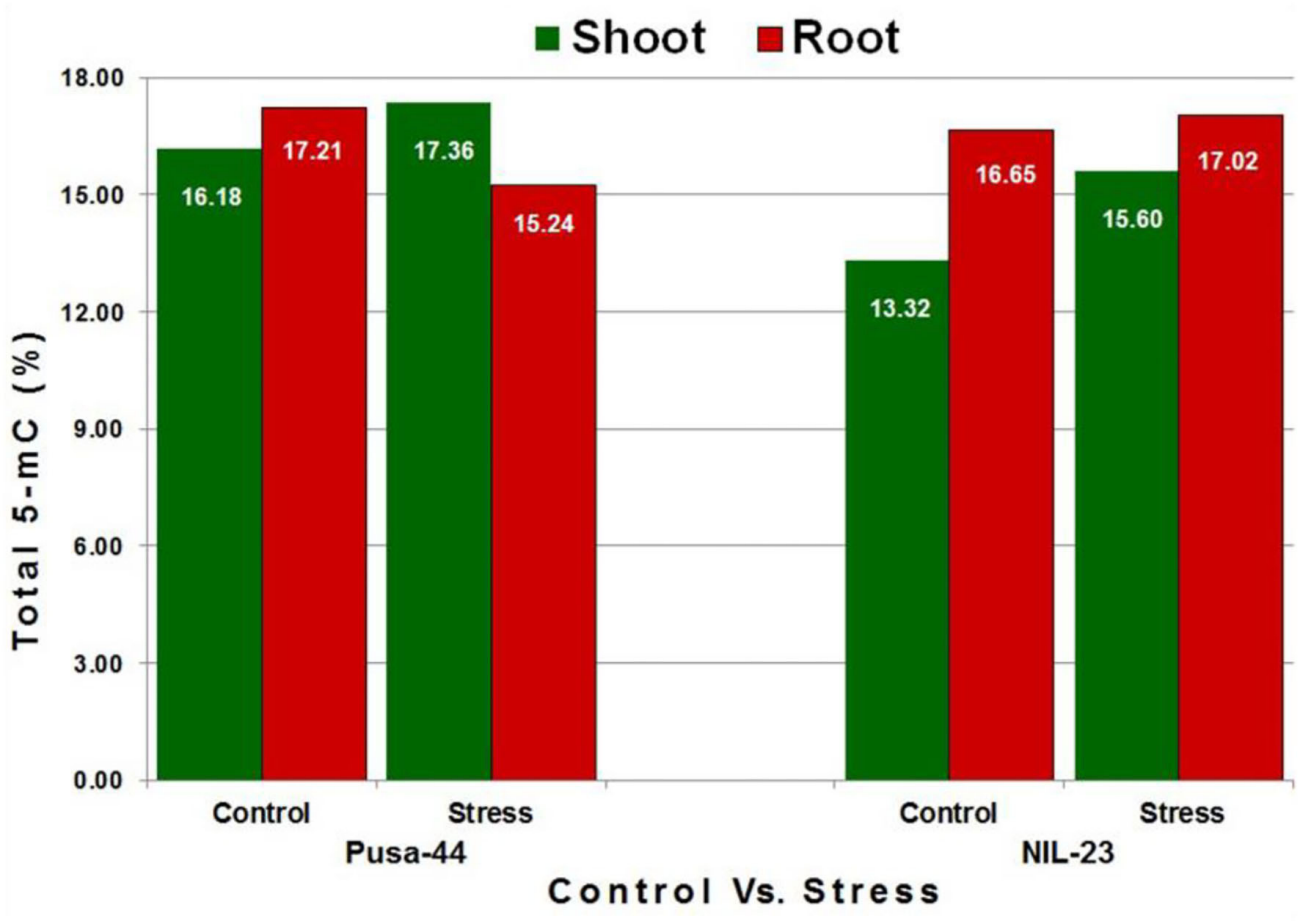
Methylcytosine (5-mC) content in shoot and root of the contrasting [(Pusa-44 stress-sensitive) and NIL-23 (stress-tolerant)] rice genotypes under control (P-sufficient, 16 ppm Pi) and stress (treatment, 0 ppm Pi). Whole-genome bisulfite sequencing was performed for shoot and root tissues, collected in three replicates, from 45-day-old rice plants grown hydroponically. 5-mC content was calculated with the help of Bismark (v0.8) software using the default parameters.

To assess the changes in DNA methylation landscape induced by P-starvation stress, genome-wide methylation profile of root and shoot tissues from 45-day-old rice plants grown under P-starvation and P-sufficient conditions was generated by sufficiently high-coverage (~25 ×), in-depth bisulfite sequencing. The average number of clean reads was 37 million, with a mapping efficiency of >50%, and the conversion rate was recorded to be >99.5% (Supplementary Table S2). Tissue- and genotype-specific variations in whole-genome 5-mC content were observed in the rice genotypes. Under the control condition, higher 5-mC content was observed in root (compared to that in the shoot) in both the genotypes. We observed 16.18% 5-mC in shoot and 17.21% in root of Pusa-44, whereas it was 13.32% in shoot and 16.65% in root of NIL-23 under the control condition. Due to the P-starvation stress, 5-mC content increased significantly in shoot, but decreased in root of Pusa-44 (stress-sensitive) genotype. In the case of NIL-23 (stress-tolerant genotype), an increase in 5-mC content was observed in shoot under the stress, but only a minor (non-significant) increase in methylation was observed in root (Figure 1).

**Figure 2:**
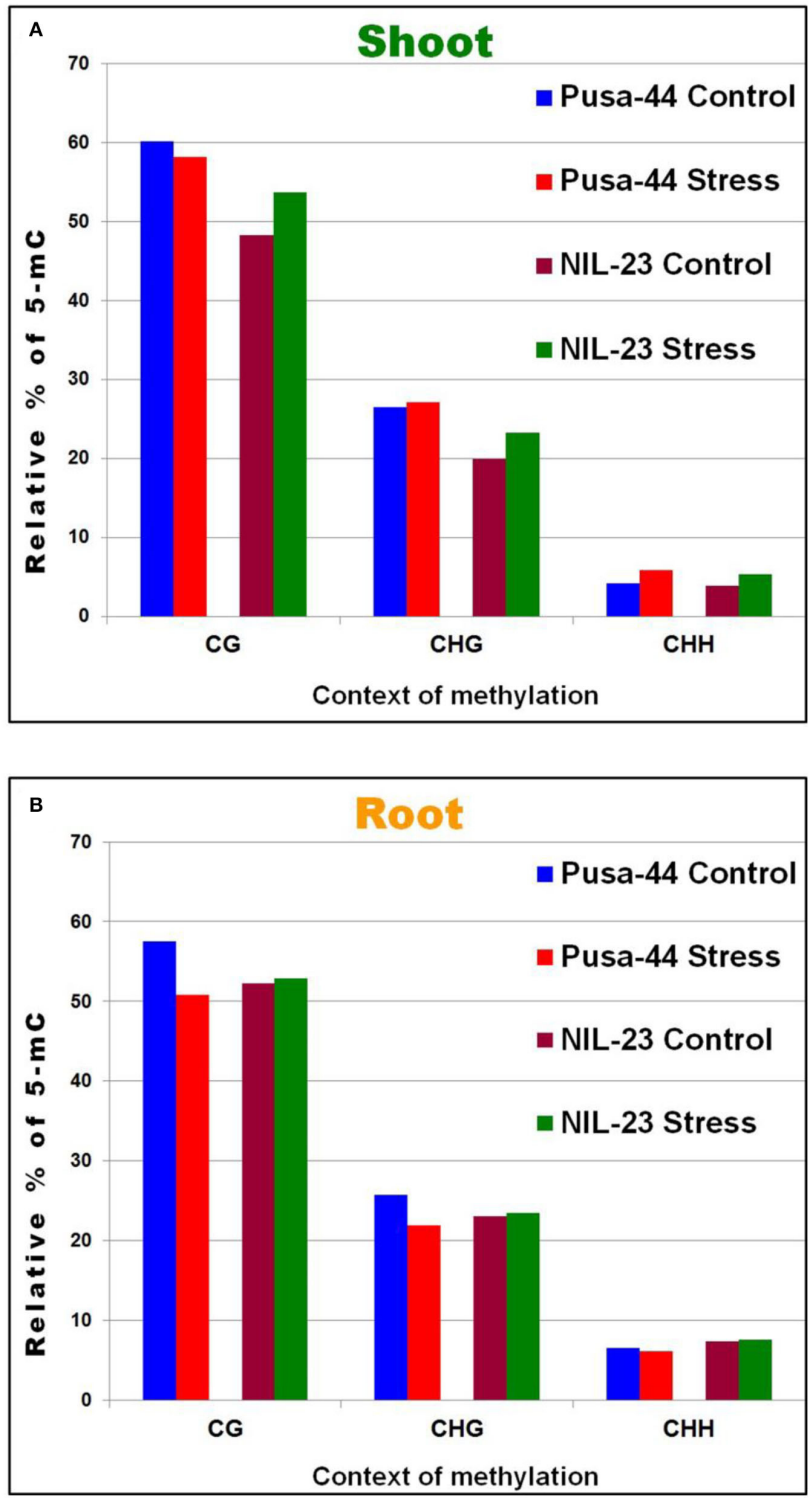
Relative distribution of DNA methylation in different (CG, GHG, and CHH) sequence contexts under P-starvation stress (treatment, 0 ppm Pi) over the control (P-sufficient, 16 ppm Pi) conditions in **(A)** shoot and **(B)** root of 45-day-old rice plants grown hydroponically.

To identify the context-specific differentially methylated cytosines (DmCs) between the control (P-sufficient) and treatment (P-starvation) at each potentially methylated cytosine, the significance of the treatment was assessed using F-test and logistic regression models with the help of Bismark software. This resulted in the observation that a significant (1.5–1.7%) increase in methylation in CHH context occurred under P-starvation stress in shoot of both the genotypes. Moreover, a significant increase in methylation was also observed in CG and CHG contexts in shoot of NIL-23 in response to the stress (Figure 2A). However, in roots of Pusa-44, a significant reduction in methylation in CG and CHG contexts, but only a marginal decrease in CHH context, was observed. A marginal (0.2–0.6%) increase in methylation in all the three (CG, CHG, and CHH) contexts was observed in response to P-starvation in root of NIL-23 (Figure 2B).

### Differential Methylation of Genomic Regions

**Figure 3:**
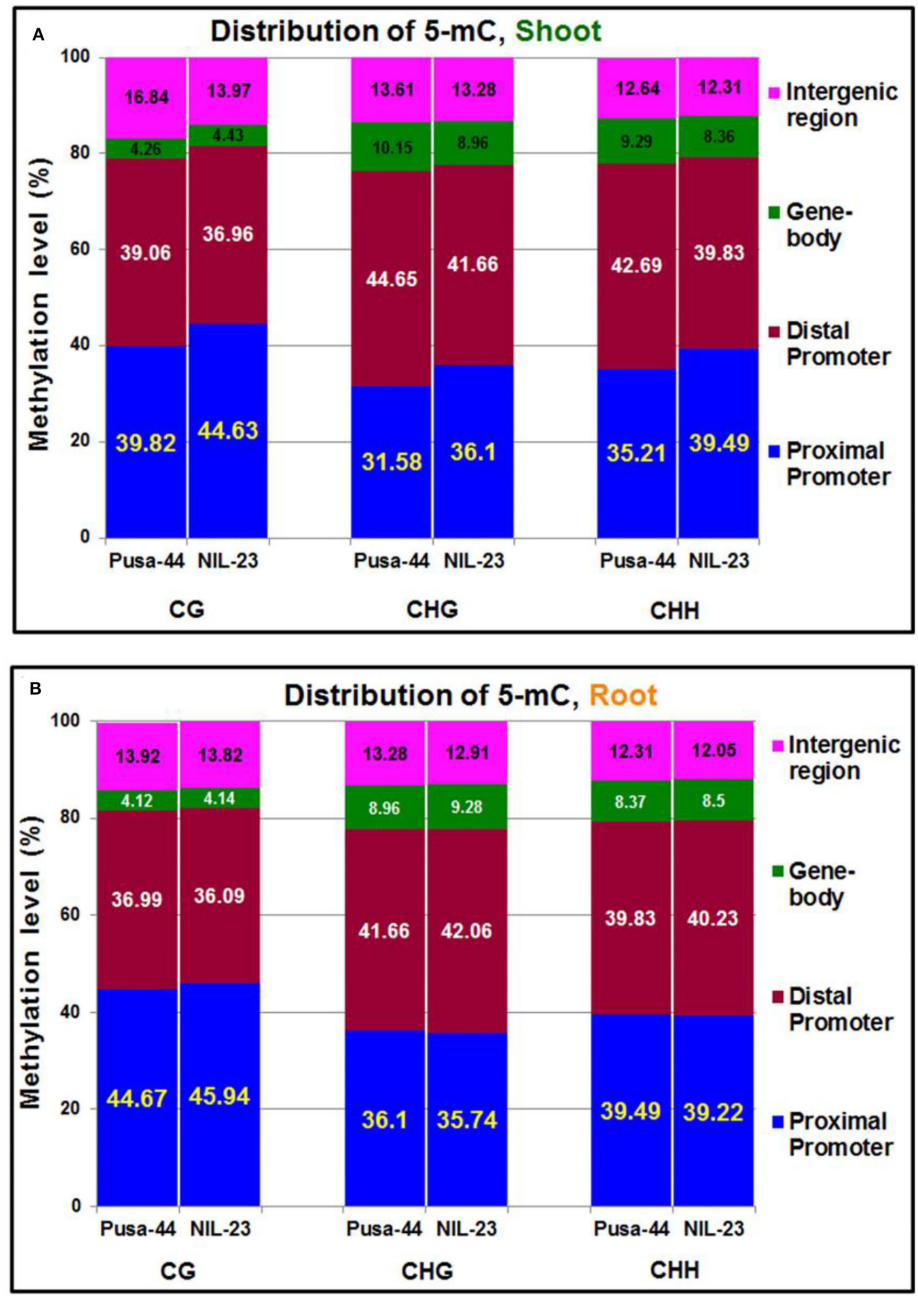
Distribution of 5-mC in different parts of the gene under treatment (P-starvation) over the control (P-sufficient) in **(A)** shoot and **(B)** root of 45-day-old rice plants grown hydroponically.

To visualize the changes in DNA methylation among different parts of the genes, the genomic regions were classified into five different regions according to the rice genome annotation: including the promoter (<1 kb, 1–2 kb, and 2–3 kb), 5′ untranslated region (UTR), gene body (1st exon, other exons, 1st intron, and other introns), 3′ UTR, and downstream region. The maximum change in DNA methylation, induced by the P-starvation stress, was observed in the promoter region. In shoot of NIL-23, 4.81% increase in methylation (i.e., 44.63%) in the proximal (<1 kb) promoter region, compared to that (39.82%) in Pusa-44, was observed in CG context. Similarly, 4.52% increase in methylation (i.e., 36.10%) in the proximal promoter region of NIL-23 shoot, compared to that (31.58%) in Pusa-44 in CHG context, and 4.28% increase in methylation (i.e., 39.49%) in CHH context in the proximal promoter region of NIL-23 shoot, compared to that (35.21%) in Pusa-44, was observed. However, a decrease in methylation in shoot of NIL-23 (compared to that in Pusa-44) was observed in the distal promoter (1–3 kb) region in all three contexts (Figure 3A). Moreover, an increase in methylation (hypermethylation) in the distal exons in CG context, but a decrease in methylation (hypomethylation) in CHG and CHH contexts, was observed in shoot of NIL-23, compared to that in Pusa-44. A decrease in methylation in all the three contexts in the distal intergenic (downstream) region was observed in shoot of NIL-23, compared to that in Pusa-44 (Figure 3A).

In root of NIL-23, only 1.27% increase in methylation (i.e., 45.94%) in the proximal (<1 kb) promoter region in CG context, compared to that (44.67%) in Pusa-44, was observed (Figure 3B). A minor (0.36%) decrease in methylation (i.e., 35.74%) in CHG context in the proximal promoter region in root of NIL-23 compared to that (36.10%) in Pusa-44, and 0.27% less methylation in CHH context in the proximal promoter region (i.e., 39.22%) in root of NIL-23 compared to that (39.49%) in root of Pusa-44, was observed under P-starvation stress. Likewise, a minor decrease (0.90%) in methylation in the distal promoter in CG context, 0.40% increase in CHG and CHH contexts in root of NIL-23 (compared to that in Pusa-44), was observed under the stress. Moreover, a marginal increase (0.02–0.32%) in methylation in all the three contexts in the gene body was observed in root of NIL-23, compared to that in Pusa-44 (Figure 3B). Furthermore, allocation of DmCs in the genomic regions revealed that about 75% of the DmCs were mapped in the promoter region, 4–8% in the gene body, and 14–17% in the intergenic regions in root of NIL-23 under the stress (Figure 3B).

We could identify 56,520 and 55,701 non-redundant DMRs (≥3 differentially methylated cytosine residues per DMR) in Pusa-44 and NIL-23, respectively, induced by the P-starvation stress. Among the DMRs, >51% were hypermethylated in Pusa-44 whereas >51% were hypomethylated in NIL-23. The highest number of DMRs (16,536, 58.12%) was hypomethylated in root of NIL-23, whereas the highest number (15,077, 51.98%) of hypermethylated DMRs was observed in shoot of Pusa-44. A higher number (24,260) of DMRs were observed in CHH context in Pusa-44 under the stress, with more (12,242) of them hypermethylated (including 6,716 in shoot). In NIL-23, a total of 21,012 (37.72%) DMRs were observed in CHH context under the stress, with more (10,920) of them hypomethylated (including 55.57% DMRs in root). The number of hypomethylated DMRs in CG (6,122), CHG (4,346), and CHH (6,068) contexts was higher than the hypermethylated DMRs [CG (5,303), CHG (3,740), and CHH (5,279)] in root of NIL-23 under the stress. Similarly, the number of hypomethylated DMRs in CG, CHG, and CHH contexts was higher than DMRs [CG (3,903), CHG (3,158), and CHH (4,852)] observed in shoot of NIL-23 under the stress.

**Figure 4:**
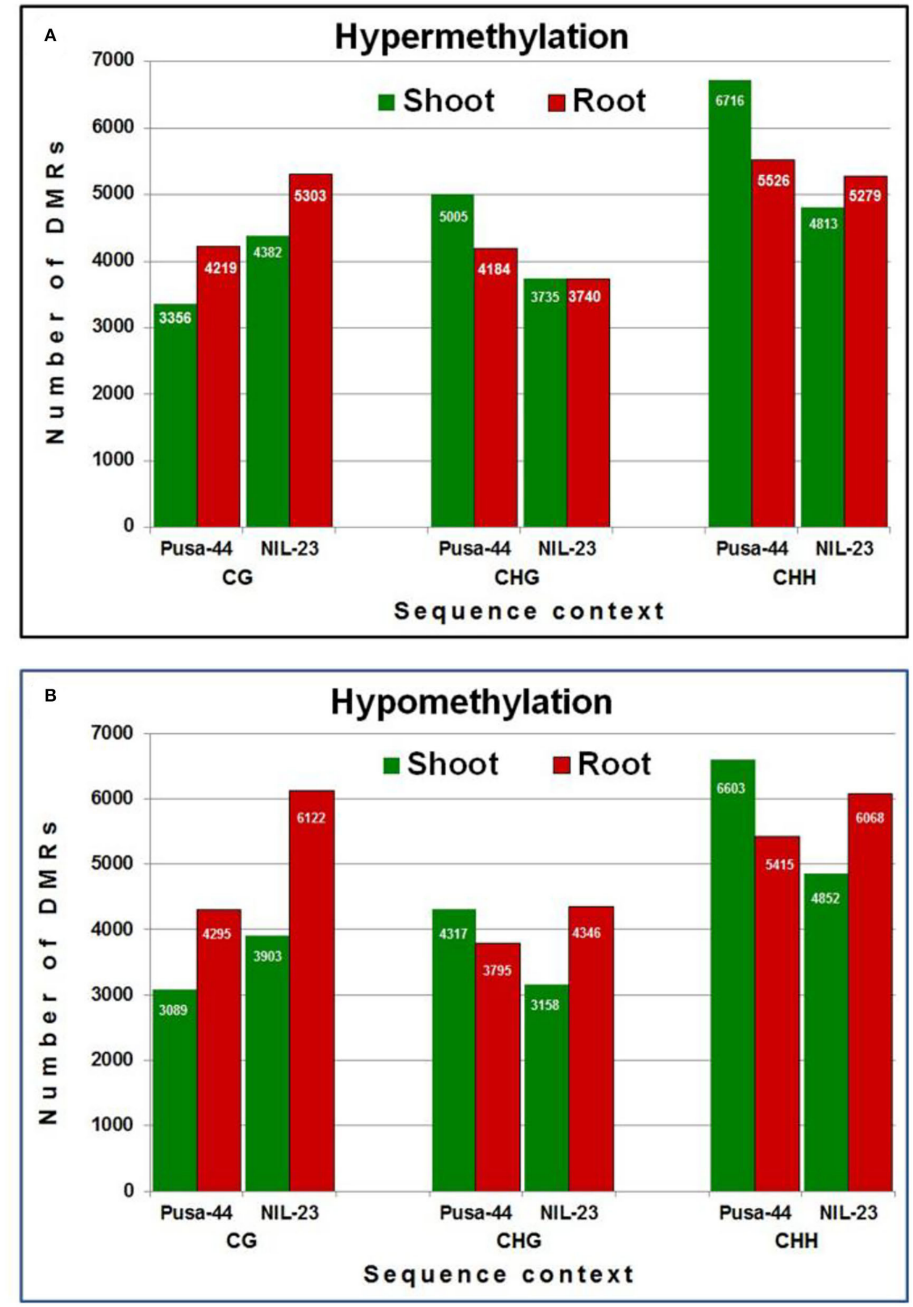
Differentially methylated regions (DMRs) in different sequence contexts of DNA in response to P-starvation stress. **(A)** Hypermethylation and **(B)** hypomethylation in shoot and root of 45-day-old rice plants of NIL-23 (stress-tolerant genotype) and Pusa-44 (stress-sensitive genotype) grown hydroponically under stress (P-starvation) analyzed over the control (P-sufficient) condition. DMRs were determined with the help of CGmapTools within 100-bp bin in the rice genome. Differentially methylated bins showing at least 20% difference in methylation level, with *Q* <0.05 (calculated using Fisher's exact test), and 2-fold change in methylation were considered as DMRs.

An increase in methylation was also observed in the CG context under the stress with more hypermethylated DMRs in shoot as well as root of NIL-23 under the stress. Nevertheless, comparatively lesser hypermethylation was observed in the CHG and CHH contexts in shoot and root of NIL-23 than that observed in Pusa-44 under the stress (Figure 4A). Comparatively more hypomethylation was observed in CG context in shoot as well as root of NIL-23 than Pusa-44 under the stress. In addition, more hypomethylation was also observed in CHG and CHH contexts in root than that in shoot of NIL-23 and root of Pusa-44 (Figure 4B).

### Distribution of DMRs in CHH Context

**Figure 5:**
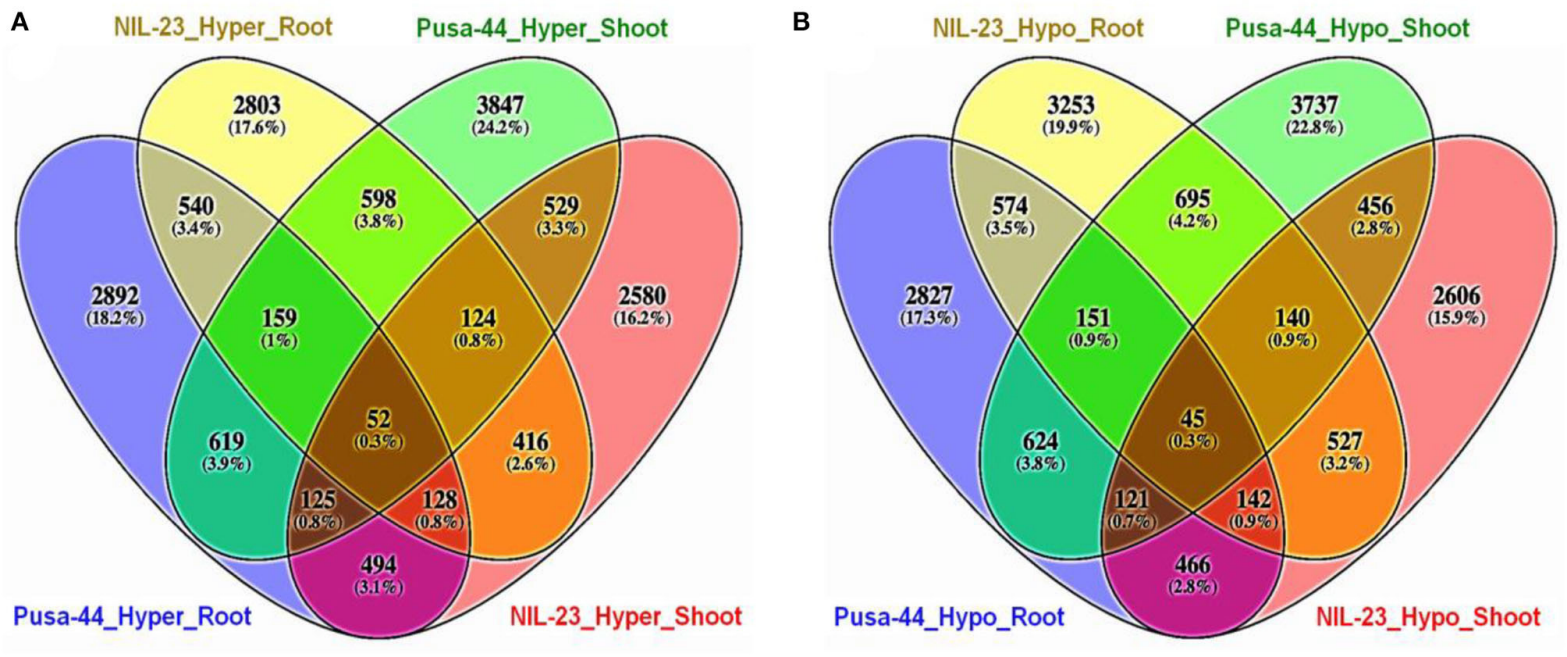
Four-way analysis of **(A)** hyper- and **(B)** hypo-methylated genes (DMRs in CHH context) in the contrasting rice genotypes (Pusa-44, P-deficiency-sensitive and NIL-23, P-deficiency-tolerant) under P-starvation stress. Differential methylation (2-fold change with *Q* <0.05) was estimated using Fisher's exact test for the rice genotypes.

Comparative analysis of the distribution of DMRs in root and shoot of the contrasting rice genotypes in CHH context indicated that the number of exclusively hypermethylated DMRs (2,803, 17.6%) in root of NIL-23 (P-deficiency-tolerant genotype) was less than that (exclusively hypermethylated DMRs 2,892, 18.2%) in root of Pusa-44. Similarly, the number of exclusively hypomethylated DMRs (3,847, 24.2%) in shoot of Pusa-44 was higher than that (exclusively hypermethylated DMRs 2,580, 16.2%) in shoot of NIL-23 (Figure 5A). On the contrary, a considerably higher number of exclusively hypomethylated DMRs (3,253, 19.9%) in CHH context were observed in root of NIL-23 compared to 2,827 (17.3%) exclusively hypomethylated DMRs in root of Pusa-44. Similarly, the number of exclusively hypomethylated DMRs (3,737, 22.8%) in shoot of Pusa-44 was higher than that (exclusively hypermethylated DMRs 2,606, 15.9%) in shoot of NIL-23 (Figure 5B).

### GO Analysis of Differentially Methylated Genes in CHH Context

**Figure 6:**
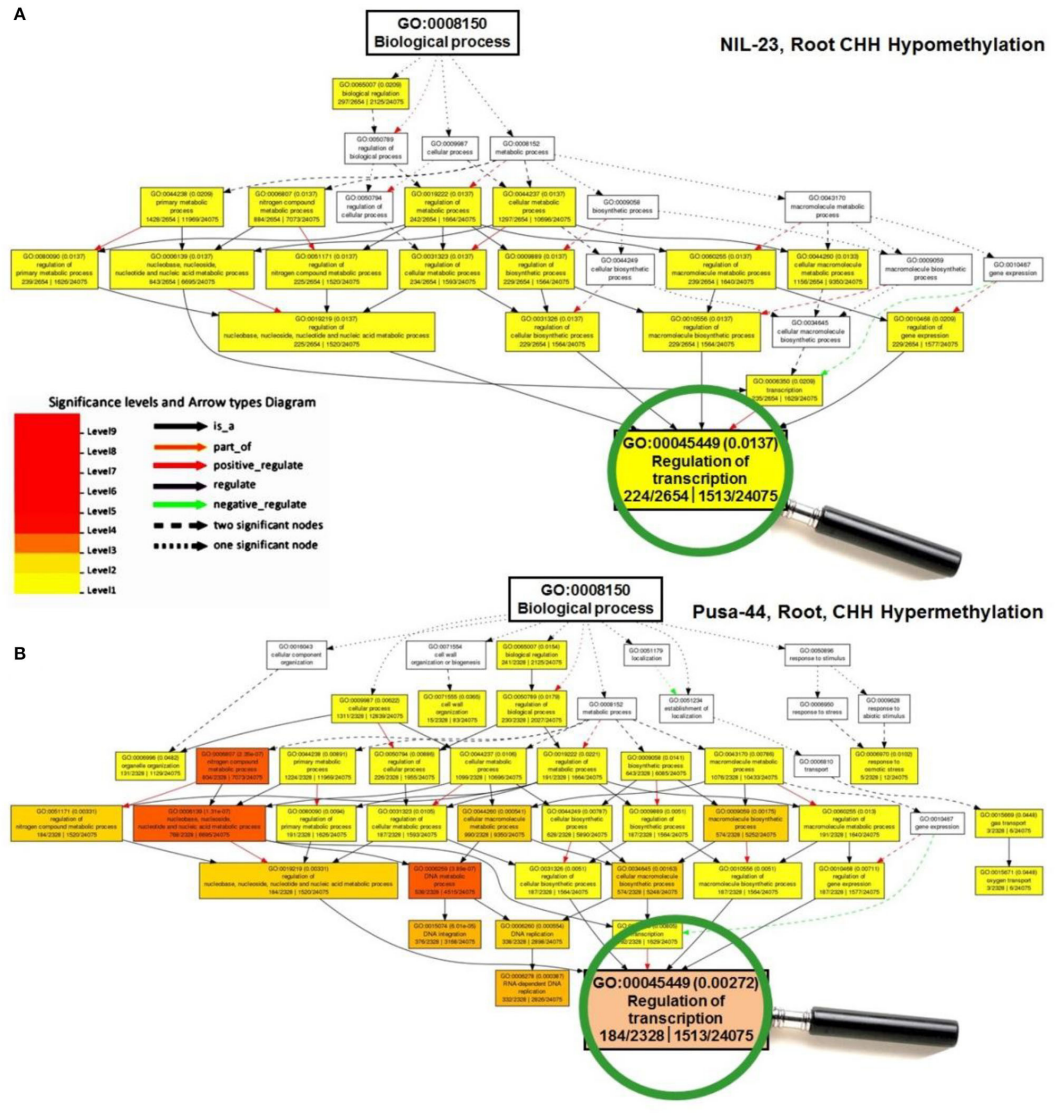
A representative figure of Gene Ontology (GO) analysis of the differentially methylated genes for GO terms of biological process under P-starvation stress in contrasting rice genotypes. **(A)** GO terms for hypomethylated genes in CHH context in root of NIL-23 (stress-tolerant genotype), and **(B)** GO terms for hypermethylated genes in CHH context in root of Pusa-44 (stress-sensitive) rice genotype. GO enrichment analysis for the differentially methylated genes was performed with the help of AgriGO v2 software using the default parameters.

To gain insights into the differentially methylated genes in CHH context and their effect on gene expression in the contrasting rice genotypes under P-starvation stress, Gene Ontology (GO) analysis was performed, which deciphered their role in modulating biological/cellular/molecular processes. GO analysis for root of NIL-23 indicated a larger number of genes to be hypomethylated in CHH context which was associated with a considerably higher number (34) of over-represented GO terms, whereas such genes were hypermethylated in CHH context in root of Pusa-44 and associated with a lesser number (27) of GO terms (Figure 6).

**Figure 7:**
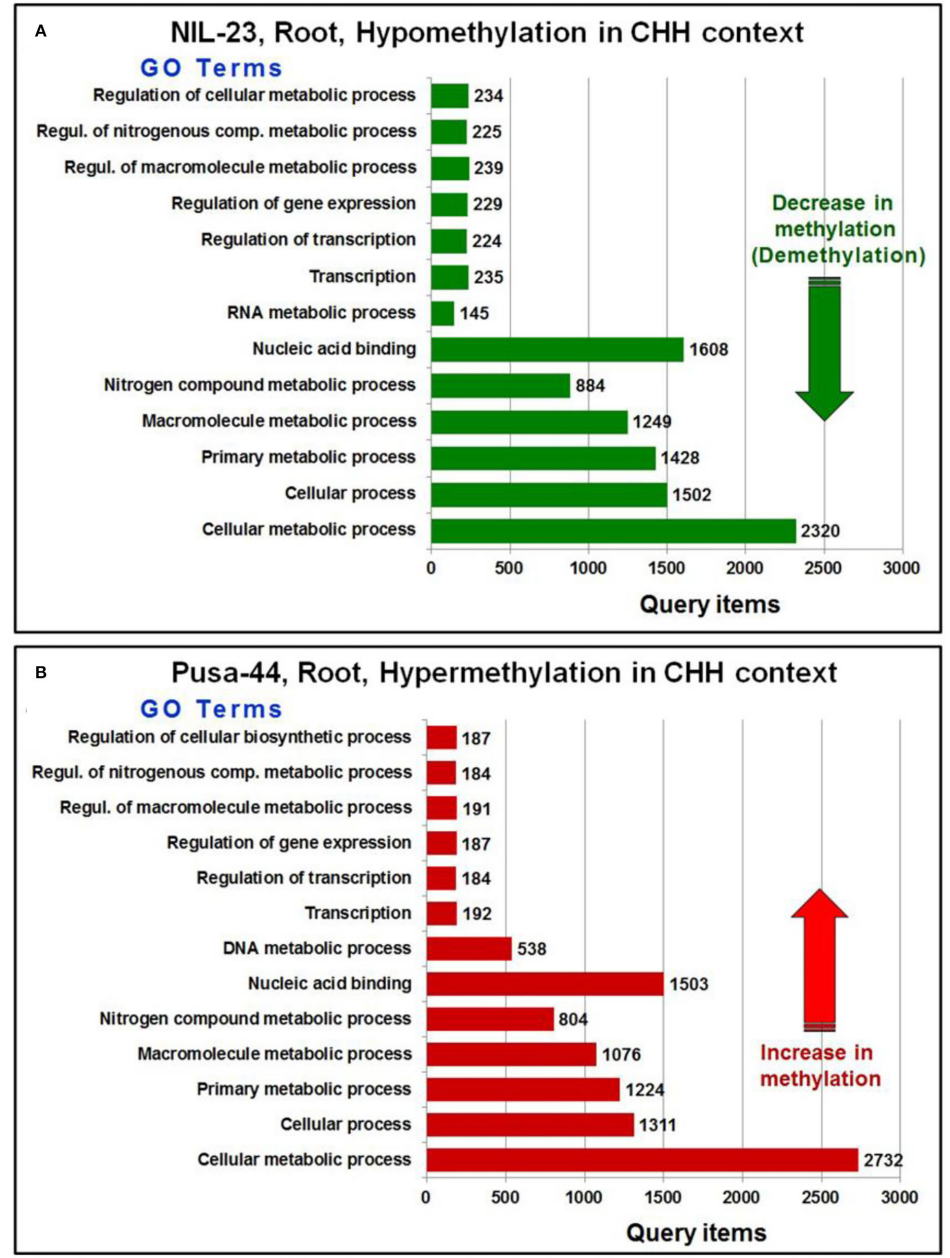
GO terms affected by changes in DNA methylation (5-mC/DMRs in CHH context) in roots of the contrasting rice genotypes (Pusa-44, P-deficiency-sensitive and NIL-23, P-deficiency-tolerant) under P-starvation stress. **(A)** The number of query items for hypomethylation in root of NIL-23 and **(B)** the number of query items for hypermethylation in root of Pusa-44. GO enrichment analysis for the differentially methylated genes was performed with the help of AgriGO v2 software using the default parameters.

Further analysis of the GO terms and the number of associated genes hypo-/hyper-methylated in CHH context in root of the contrasting rice genotypes indicated that a large number of genes were differentially methylated (hypomethylated in root of NIL-23, while hypermethylated in root of Pusa-44) in the rice genotypes (Figure 7). A considerably large number of genes for the GO terms cellular processes and cellular metabolic process were hypomethylated in CHH context in root of NIL-23 (stress-tolerant genotype), whereas the genes were hypermethylated in root of Pusa-44 (stress-sensitive genotype) under the stress. Similarly, genes for the GO terms primary metabolic process, macromolecule metabolic process, nitrogenous compound metabolic process, and nucleic acid-binding proteins were hypomethylated in root of NIL-23, but hypermethylated in root of Pusa-44 under the stress.

Moreover, genes for the GO terms regulation of macromolecule metabolic process, regulation of the cellular metabolic process, regulation of nitrogenous compound metabolic process, regulation of gene expression, and regulation of transcription were hypomethylated in CHH context in root of NIL-23, whereas the genes for these GO terms were hypermethylated in root of Pusa-44 under the stress. Furthermore, genes for the GO terms transcription and RNA metabolic processes were hypomethylated in CHH context in root of NIL-23 (Figure 7A), whereas the genes for transcription and DNA metabolic processes were hypermethylated in CHH context in root of Pusa-44 (Figure 7B).

### Effect of DNA Methylation in CHH Context on Gene Expression

**Figure 8:**
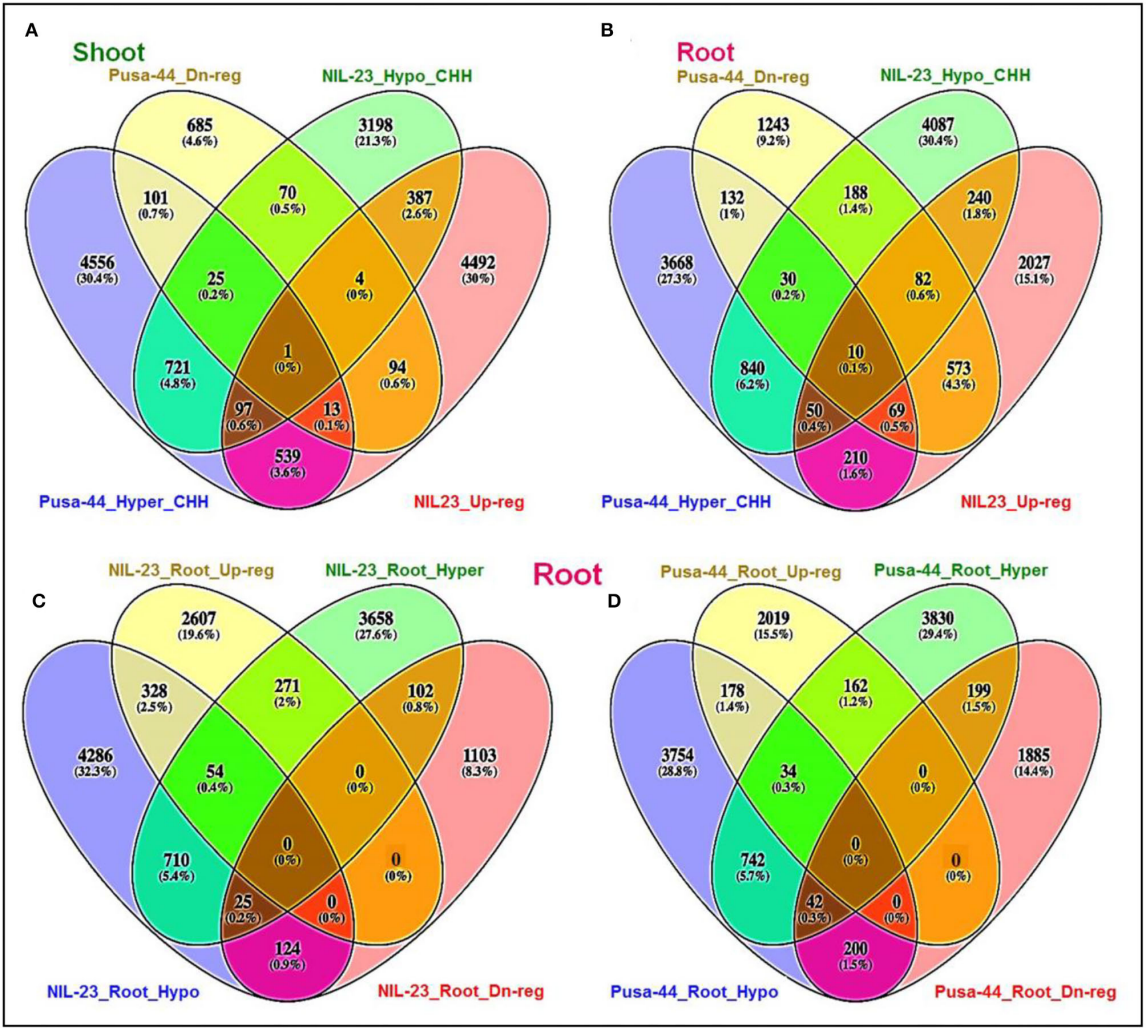
Epigenetic regulation of gene expression though hyper- or hypomethylation of genes in CHH context in the contrasting rice (NIL-23, P-deficiency-tolerant and Pusa-44, P-deficiency-sensitive) genotypes grown under P-starvation stress. **(A)** Hypo-/hypermethylation causing up-/downregulated expression in shoot of contrasting rice genotypes, **(B)** hypo-/hypermethylation causing up-/downregulated expression in root, **(C)** hypo-/hypermethylation causing up-/downregulated expression in root of NIL-23, **(D)** hypo-/hypermethylation causing up-/downregulated expression in root of Pusa-44. The correlation between differential methylation (2-fold change with *Q* <0.05) and differential gene expression (2-fold change with *p* < 0.05) under P-starvation stress over the control condition was estimated for the tissues and rice genotypes.

Hypomethylation (decrease in methylation) of DNA in CHH context caused upregulated expression of 489 genes in shoot of NIL-23, whereas 140 of the genes were hypermethylated and downregulated in Pusa-44. Among these hypomethylated genes, 387 genes were upregulated exclusively in shoot of NIL-23 (Figure 8A). Similarly, hypomethylation in the CHH context caused upregulated expression of 382 genes in root of NIL-23, whereas 241 of the genes were hypermethylated and downregulated in Pusa-44. Among these hypomethylated genes, 240 genes were upregulated exclusively in root of NIL-23 (Figure 8B). Thus, hypomethylation in CHH context, particularly in the promoter region, in root of NIL-23 caused upregulated expression of the gene, whereas hypermethylation of the genes in the CHH context in root of Pusa-44 caused downregulated expression. About 3.9% of the genes hypo- or hyper-methylated in CHH context in roots of NIL-23 could be correlated with the expression level of the genes, whereas it was ~1.8% in the case of Pusa-44 (Figure 8). Many of the P-responsive genes, some of the genes for P transporters, transcription factors (TFs), and those involved in epigenetic modifications (DNA methylation as well as histone modification) were observed to be regulated through modulation in cytosine methylation (Supplementary Table S3).

**Figure 9:**
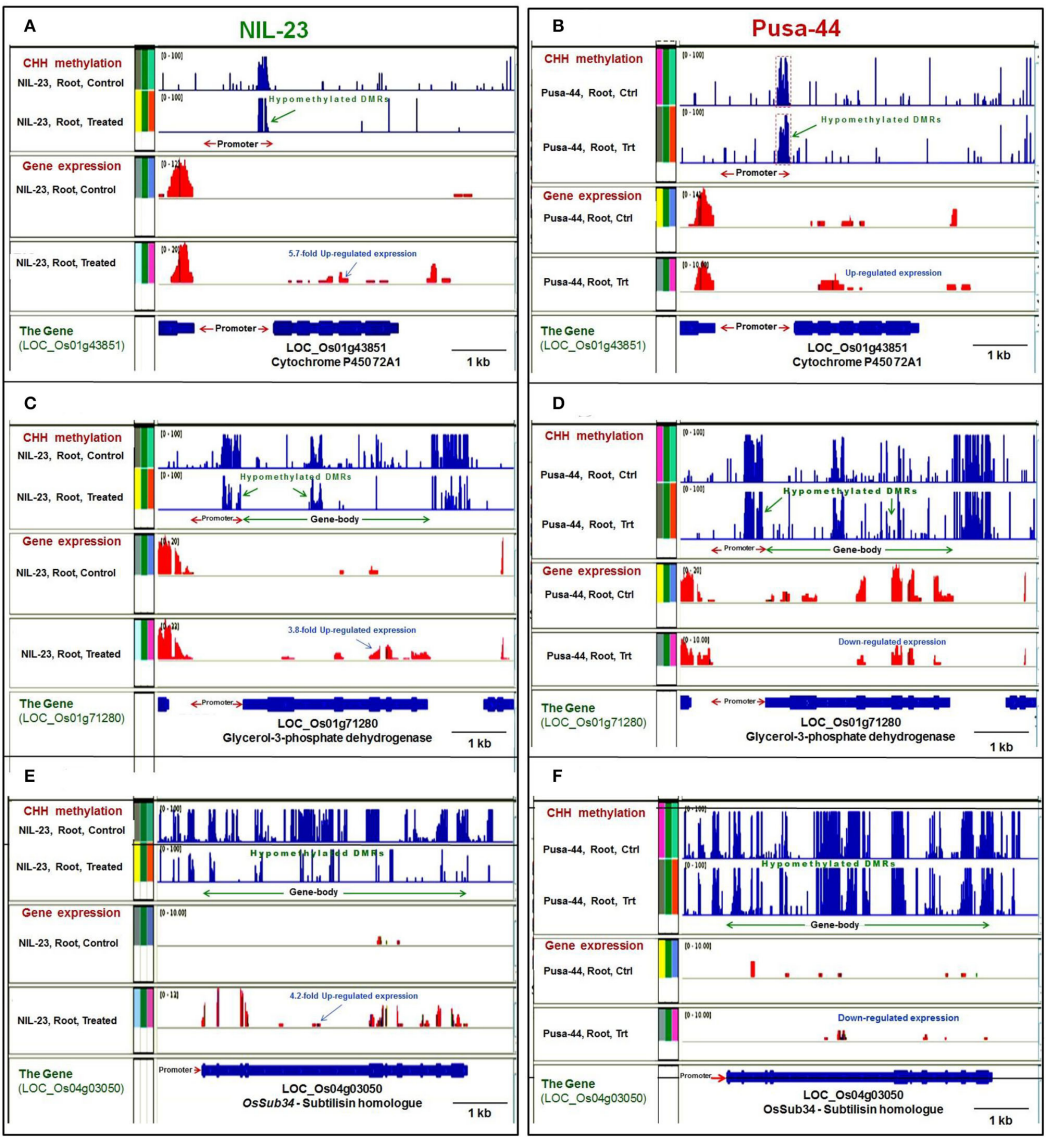
Representative genome browser screenshots showing the effects of DNA methylation on gene expression by hyper- or hypomethylation of gene in CHH context in contrasting rice (NIL-23, P-deficiency-tolerant and Pusa-44, P-deficiency-sensitive) genotypes grown hydroponically under control (16 ppm Pi) and P-starvation (0 ppm Pi) stress. **(A)** Hypomethylation of (proximal) promoter caused upregulated expression of cytochrome P450 72A1 gene in root of NIL-23 under the stress, **(B)** lesser hypomethylation caused lesser upregulated expression of the gene in root of Pusa-44. **(C)** Hypomethylation of the (proximal) promoter, as well as the gene body, caused upregulated expression of LOC_Os01g71280 in root of NIL-23, **(D)** lesser hypomethylation of promoter and gene body caused downregulated expression of the gene in root of Pusa-44 under the stress. **(E)** Hypomethylation of gene body caused upregulated expression of LOC_Os04g03050 in root of NIL-23, **(F)** lesser hypomethylation caused downregulated expression of the gene observed in root of Pusa-44 under the stress. The blue bars (in upper 2 panels) represent methylation level (DMRs), and the expression level of gene is represented with red bars. Ctrl, control, Trt, P-starvation stress treatment.

Correlation analysis for methylation in different parts of the gene [location of differentially methylated cytosines (DmCs)] and the gene expression level indicated that most of the genes were epigenetically regulated through (de)methylation of the promoter, particularly in the proximal (<1 kb) promoter region (Figure 9, Supplementary Table S4). Hypermethylation in the proximal promoter region had a greater inhibitory effect on the expression of the gene, whereas methylation of the distal promoter (1–2 or 2–3 kb) had a lesser effect on the repression of gene expression. Moreover, methylation of gene body (particularly exon) had even stimulated effects on the gene expression.

In root of NIL-23, 382 (2.9%) genes were observed to be upregulated due to hypomethylation in the CHH context, whereas 127 (1.0%) genes were downregulated due to hypermethylation in the CHH context (Figure 8C). Such correlation between gene expression and DNA methylation (212 genes upregulated due to hypomethylation and 241 genes downregulated due to hypermethylation in CHH context) in root of Pusa-44 (Figure 8D) was significantly different from that in NIL-23. Thus, a lesser number of genes were hypomethylated or upregulated in root of Pusa-44, whereas a higher number of genes were hypermethylated or downregulated in root of Pusa-44 compared to that in NIL-23. Interestingly, these changes in NIL-23 were brought in due to the introgression of *Pup*1 QTL.

Detailed analysis of the hypomethylated (in CHH context) and upregulated genes indicated that many of the P-responsive genes such as serine–threonine protein kinase, phosphoesterase, phosphoglycerate kinase, glycerol-3-phosphate dehydrogenase, dirigent, expansin, phosphate-induced protein 1, purple acid phosphatase, monogalactosyldiacylglycerol synthase 2, amino acid transporters, and BAG domain-containing protein, and so on. were upregulated in roots of NIL-23 due to hypomethylation or demethylation under P-starvation stress (Supplementary Table S5). A large number of genes, including Shikimate kinase (catalyzes the ATP-dependent phosphorylation of shikimate to form shikimate 3-phosphate), sucrose-phosphate synthase, cyclin-dependent kinase, phosphoribosyl transferase, phosphatidylinositol-4-phosphate 5-kinase, phosphatidylinositol kinase, ATPase, fructose-1,6-bisphosphatase, glyceraldehyde-3-phosphate dehydrogenase, hexokinase, ATP-dependent RNA helicase, DNA-directed RNA polymerase I, phosphoribosyl transferase, photosystem I reaction center subunit, and aminotransferases, etc., were hypermethylated and downregulated in root of NIL-23 compared to that under control conditions (Supplementary Table S6).

Moreover, the genes hypomethylated in CHH context resulting in their upregulated expression in root of Pusa-44 included those for glycosyltransferase, group 1 domain-containing protein, phosphoethanolamine or phosphocholine phosphatase, glutathione S-transferase, U-box domain-containing protein, thiamine pyrophosphate enzyme, senescence-induced receptor-like serine–threonine protein kinase precursor, nucleotide pyrophosphatase or phosphodiesterase, and thiol protease SEN102 precursor, etc. (Supplementary Table S7), which were different from those hypomethylated in root of NIL-23 under the stress. Similarly, a large number of genes hypermethylated and downregulated in root of Pusa-44 included those for fructose-bisphosphate aldolase isozyme, MYB family TF, BSD domain-containing protein, S-adenosylmethionine synthetase, phosphoesterase family protein, 60S ribosomal protein L37a, phosphatase family domain-containing protein, amino acid kinase, auxin response factor, DEAD-box ATP-dependent RNA helicase, plastidic ATP/ADP-transporter, NHL repeat-containing protein, coiled-coil domain-containing protein, serine–threonine protein kinase, phenylalanine ammonia-lyase, helix–loop–helix DNA-binding protein, DNA-binding protein, ethylene-responsive transcription factor, glycosyl hydrolases family 17, kinesin-related protein, stress-responsive protein, phosphoglyceratemutase, CTP synthase, and zinc finger family protein, etc., which were largely different from those hypermethylated and downregulated in root of NIL-23 (Supplementary Table S8).

Hypomethylation at a proximal promoter region was observed to cause upregulated expression of genes, e.g., cytochrome P450 72A1, in root of NIL-23 under the stress (Figure 9A), which was less hypomethylated and less upregulated in root of Pusa-44 (Figure 9B). Similarly, hypomethylation of promoter caused upregulated expression of a gene encoding protein kinase domain-containing protein in root of NIL-23 under the stress (Supplementary Figure S1A), whereas no significant change in methylation of the promoter, but a downregulated expression of the gene, was observed in root of Pusa-44 under the stress (Supplementary Figure S1B). Hypomethylation at proximal promoter as well as the gene body caused upregulated expression of LOC_Os01g71280 in root of NIL-23 under the stress (Figure 9C) whereas hypomethylation of promoter and gene body caused downregulated expression of the gene in root of Pusa-44 under the stress (Figure 9D). Likewise, hypomethylation of promoter as well as the coding region (gene body) of the gene for serine–threonine protein kinase caused upregulated expression of the gene in root of NIL-23 under the stress (Supplementary Figure S1C), but hypermethylation of promoter, as well as the gene body, caused downregulated expression of the gene in root of Pusa-44 under the stress (Supplementary Figure S1D). Some of the genes hypomethylated in CHH context in the gene body (e.g., LOC_Os04g03050) were observed to be upregulated in root of NIL-23 under the stress (Figure 9E), but the gene (LOC_Os04g03050) was hypomethylated and downregulated in root of Pusa-44 (Figure 9F). In contrast, some other genes (e.g., LOC_Os02g19924) were hypermethylated in CHH context in the gene body and upregulated in root of NIL-23 under the control conditions (Supplementary Figure S1E), but hypomethylation of the gene under the stress could not result in any change in its expression level in root of Pusa-44 (Supplementary Figure S1F).

### Effect of DNA Methylation on Expression of Transcription Factors

Several TFs were found to be differentially expressed in the contrasting rice genotypes under P-starvation stress. Many of the TFs, including HSF-type DNA-binding domain-containing protein, MYB family transcription factors, no apical meristem protein, homeobox-associated leucine zipper, helix–loop–helix DNA-binding domain-containing protein, and OsMADS, etc., were observed to be upregulated due to hypomethylation in the promoter region. Some of the TFs (e.g., ethylene-responsive transcription factor 2, MYB family transcription factor LOC_Os01g08160) were downregulated in root of NIL-23 (compared to their expression under control conditions and in root of Pusa-44 under the stress) due to increased methylation (DMR), mainly in the promoter (Supplementary Table S9).

### Effect of DNA Methylation in CHG Context on Gene Expression

A large number (>197) of genes hypomethylated in CHG context in the promoter region (and upregulated) in root of NIL-23 were observed to be hypermethylated (downregulated) in root of Pusa-44. Among these hypomethylated or demethylated genes, 56 genes were demethylated exclusively in root of NIL-23, whereas 141 genes were hypermethylated, including 54 genes >2-fold hypermethylated (Supplementary Table S10). Some of the important genes were hypomethylated in the CHG context and showed upregulated expression in root of NIL-23, but hypermethylated and downregulated in root of Pusa-44, which included AP2 domain-containing protein, serine carboxypeptidase, PHD-finger domain-containing protein, and glutathione S-transferase. Certain genes such as NAC domain transcription factor, SUR4 membrane family protein, multicopper oxidase domain-containing protein, profilin domain-containing protein, gibberellin 20 oxidase 2, SPX domain-containing protein, cyclin-dependent kinase inhibitor, and MGD2, and so on. were upregulated even after >2-fold hypermethylation in CHG context in root of Pusa-44, whereas expression of these genes showed negative correlation with methylation level in root of NIL-23 (Supplementary Table S10). Interestingly, such change in the regulation of gene expression in NIL-23 was brought in due to introgression of *Pup*1 QTL.

Gene Ontology enrichment analysis of the gene clusters promoted for their expression due to DNA hypomethylation (demethylation in CHG context in promoter) in root of NIL-23 under P-starvation stress indicated more (double) number of genes in the clusters, particularly for catalytic activity, cellular metabolic process, primary metabolic process, and membrane-bound organelle clusters, etc. (Supplementary Figures S2A,B). Similarly, the gene clusters repressed for their expression due to DNA hypermethylation (methylation in CHG context in promoter) in root of Pusa-44 under P-starvation stress indicated more (double) number of genes in the clusters, particularly for carbohydrate metabolic process, cellular metabolic process, membrane, and intracellular organelle, etc. (Supplementary Figures S2C,D). Moreover, the gene clusters repressed for their expression due to DNA hypermethylation in shoot of Pusa-44 under P-starvation stress indicated more (double) number of genes in the clusters, particularly for developmental process, regulation of cellular process, biological regulation, and nucleus, etc. (Supplementary Figures S2G,H).

### Effect of DNA Methylation in CG Context on Gene Expression

**Figure 10:**
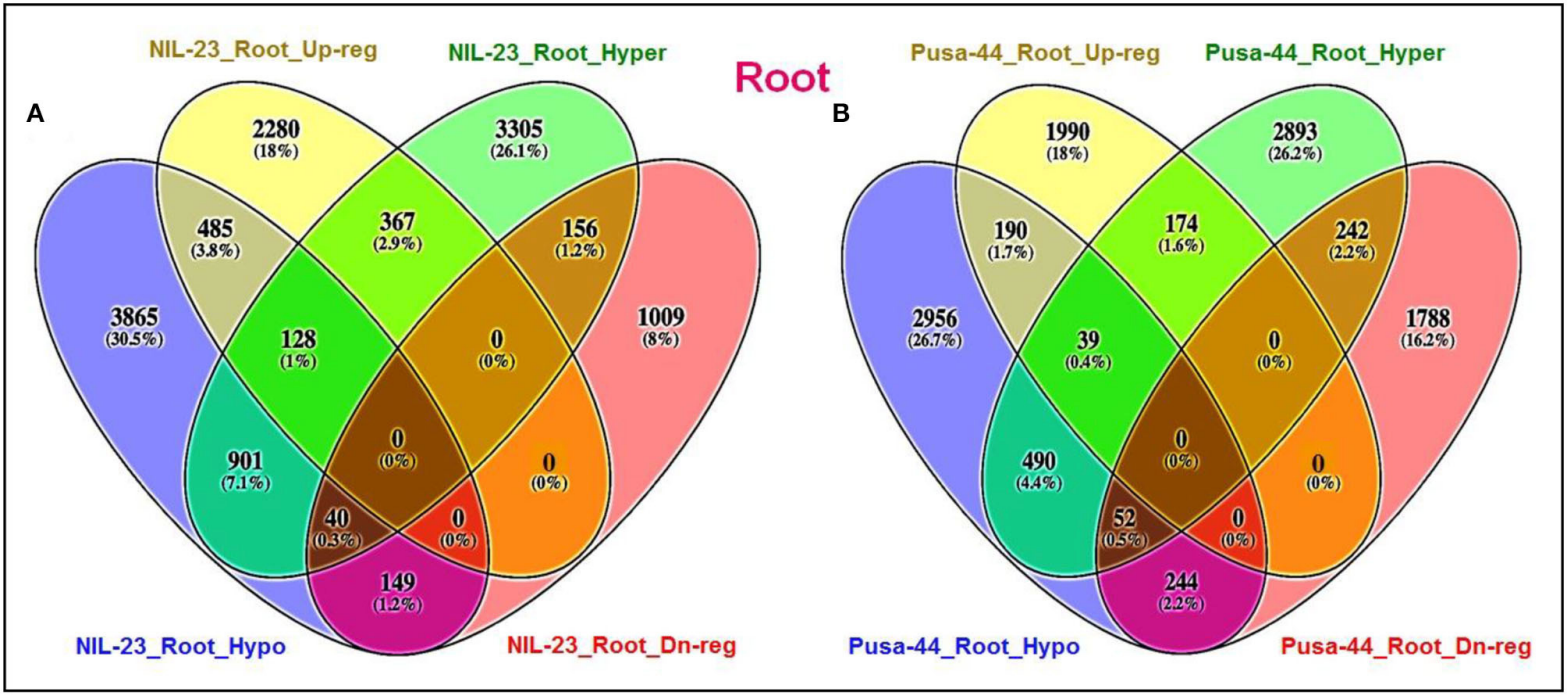
Epigenetic regulation of gene expression though hyper- or hypomethylation of genes in CG context in the contrasting rice (NIL-23, P-deficiency-tolerant and Pusa-44, P-deficiency-sensitive) genotypes grown under P-starvation stress. **(A)** Hypo-/hypermethylation causing up-/downregulated expression in root of NIL-23, **(B)** hypo-/hypermethylation causing up-/downregulated expression in root of Pusa-44. The correlation between differential methylation (2-fold change with *Q* <0.05) and differential gene expression (2-fold change with *p* <0.05) under P-starvation stress over the control condition was estimated for the rice genotypes.

Hypomethylation of DNA in CG context caused upregulated expression of 613 genes in root of NIL-23, whereas 196 genes were hypermethylated and downregulated in root of NIL-23 (Figure 10). Many multicopper oxidase (MCO) genes (e.g., LOC_Os08g39390, LOC_Os05g11660, LOC_Os09g36950, LOC_Os03g47420, etc.) were observed to be upregulated either because of hypomethylation or due to hypermethylation in CG context in root of NIL-23 (Supplementary Tables S11, S12). Interestingly, 367 (2.9%) genes were hypermethylated in CG context and showed upregulated expression in root of NIL-23 (Figure 10A). Similarly, hypomethylation of DNA in CG context caused upregulated expression of 229 genes in root of Pusa-44, whereas 294 genes were hypermethylated and downregulated in root of Pusa-44 (Figure 10B, Supplementary Tables S13, S14). Interestingly, 174 genes were hypermethylated in CG context, particularly in the gene body, and showed their upregulated expression in root of Pusa-44 (Figure 10B).

**Figure 11:**
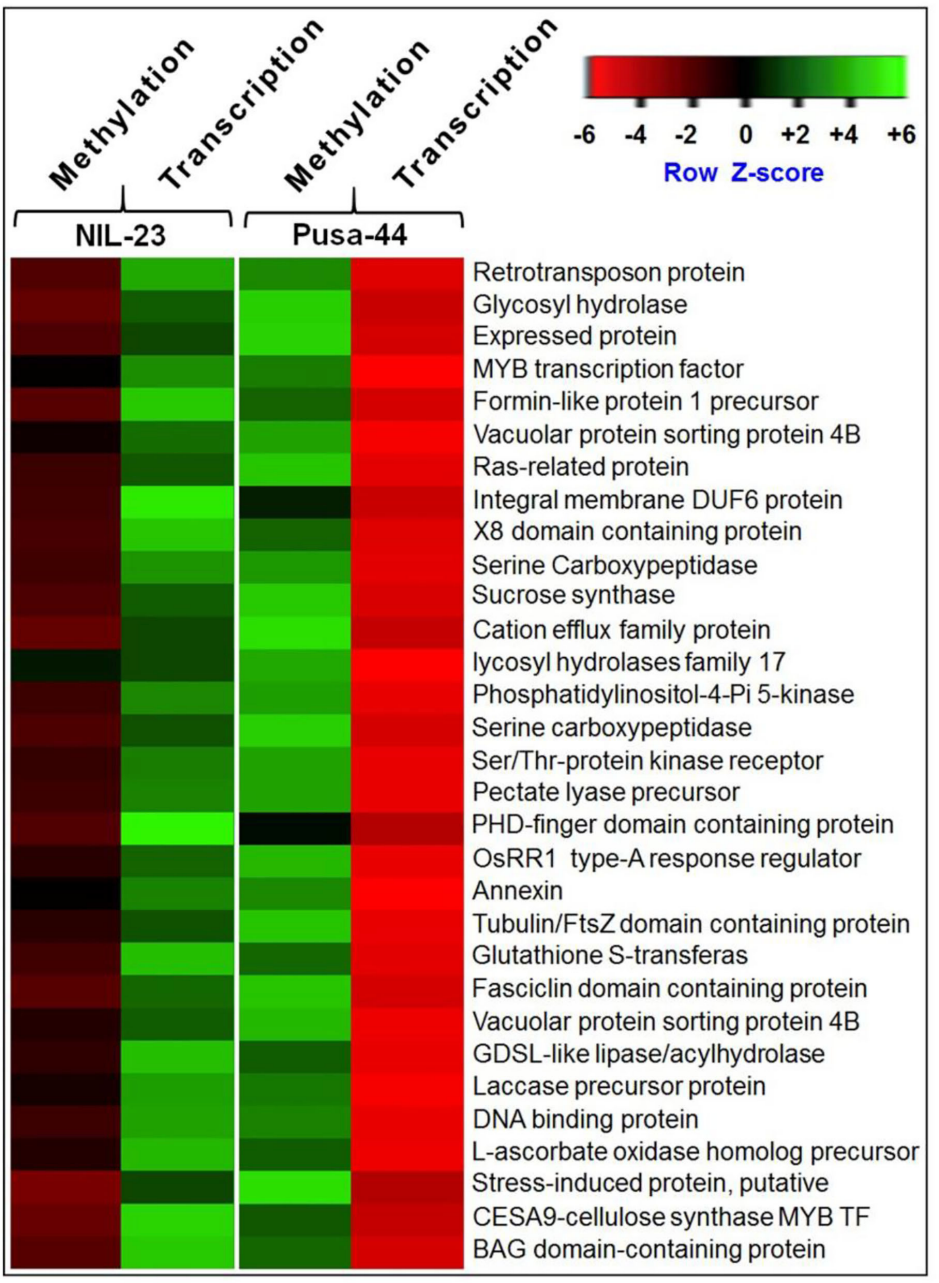
Heat map showing differential methylation in CG context and its effect on gene expression in root of the contrasting rice (NIL-23, P-deficiency-tolerant and Pusa-44, P-deficiency-sensitive) genotypes grown under P-starvation stress.

About 5.0% of the genes showing epigenetic variation (hypo- or hypermethylation) in CG context showed their effect on gene expression in roots of NIL-23 (Figure 10A), whereas it was ~3.9% in the case of Pusa-44 (Figure 10B). Many of the genes hypomethylated and upregulated in root of NIL-23 were observed to be hypermethylated and downregulated in root of Pusa-44 (Figure 11). However, the location of DMR in some of the genes was observed to be different in root of NIL-23 and Pusa-44 (Supplementary Table S15). Some of such important genes included those coding for MYB transcription factor, serine–threonine protein kinase receptor, PDH-finger domain-containing protein, annexin, tubulin/FtsZ domain-containing protein, vacuolar protein sorting—protein 4B, DNA-binding protein, stress-induced protein, and BAG domain-containing protein, etc. More interestingly, some of the unannotated, expressed genes were also observed to be hypermethylated and downregulated in root of NIL-23 but hypomethylated and upregulated in root of Pusa-44 (Supplementary Table S16).

### Effects of Gene Body Methylation

Hypermethylation in CHH context in the gene body (exon and intron) caused up- or downregulated expression of some of the genes such as terpene synthase, transmembrane protein 136, aminotransferase, sucrose synthase, potassium, and sulfate transporters, and so on (Supplementary Table S17). The downregulated genes because of hypermethylation in CHH context in the gene body included the translation initiation factor SUI1, vacuolar ATP synthase 98 kDa subunit, acyl-desaturase, and phosphoribosyl aminoimidazole carboxylase, etc. Analysis of DNA methylation in CG context in the gene body (exon and intron) indicated a large number of genes to be up- or downregulated due to hypermethylation (Supplementary Table S18). Most of the genes upregulated due to hypermethylation in CG context, generally in the exons, included the glycosyltransferases, phosphoesterases, WRKY80, gibberellin 20 oxidase 2, carboxyl-terminal peptidase, phospholipase, integral membrane protein, and retrotransposon protein, etc. However, the genes downregulated due to hypermethylation in the CG context included the protein-binding polypeptide, RNA methyltransferase, monolignol beta-glucoside homolog, amino acid transporter, and glutamate receptor, etc. Thus, hypermethylation of genes at gene body may cause up- or downregulation, which included the genes coding for TFs, transporters, phosphoesterases, retrotransposon proteins, and many other expressed proteins (Supplementary Table S18).

### Expression of Genes for DNA (De)Methylation Under P-Starvation Stress

The expression level of genes for maintaining CG methylation (LOC_Os03g58400, LOC_Os09g27060, LOC_Os03g51230), CHG methylation (LOC_Os10g01570, LOC_Os03g12570), CHH methylation (LOC_Os11g01810, LOC_Os12g01800), DNA glycosylation (LOC_Os01g11900), and RNA-dependent RNA polymerase (required for RNA-directed DNA methylation, LOC_Os01g10130) was observed to be upregulated in root of NIL-23, whereas most of them were downregulated in root of Pusa-44. Some of the genes for maintaining CG methylation (LOC_Os07g08500), CHG methylation (LOC_Os05g13780), CHH methylation (LOC_Os03g02010, LOC_Os05g04330, LOC_Os01g42630), DNA glycosylation(LOC_Os05g37410, LOC_Os02g29380, LOC_Os05g37350), and RNA-dependent RNA polymerase required for RNA-directed DNA methylation (LOC_Os01g10140, LOC_Os02g50330, LOC_Os04g39160, LOC_Os01g34350) were detected to be downregulated in root of NIL-23, but less downregulated or even upregulated in root of Pusa-44 under the stress (Supplementary Table S19).

### Epigenetic Regulation of the Regulators

Analysis of the changes in cytosine (de)methylation level in the genes coding for DNA methylases or demethylases and RDR polymerases indicated that 5-mC in different promoter regions has a significant repressive effect on gene expression. Hypermethylation of promoter caused downregulated expression of the gene. However, methylation at the proximal promoter region had more impact on the downregulation of the gene, compared to that caused by the methylation of the distal promoter region (Supplementary Table S20). Interestingly, methylation of gene body (exon) could upregulate (e.g., LOC_Os03g12570) or downregulate (e.g., LOC_Os02g29390) the gene expression. When methylation of promoter combined with gene body, they had combined effects. When methylation of exon caused upregulated expression of a gene (e.g., LOC_Os03g12570), its combination with methylation of promoter caused lowered expression of the gene (mild repressive effect). Similarly, methylation of exon caused downregulated expression of genes (e.g., LOC_Os05g04330) but its combination with methylation of the promoter caused further downregulated expression of the gene (severe repressive effect) (Supplementary Table S19). More interestingly, variation in the location of DMRs, genic regions containing DMRs, and their effects on the gene expression was observed among the rice genotypes (Supplementary Table S20).

### Validation of the Expression Level of Genes

**Figure 12:**
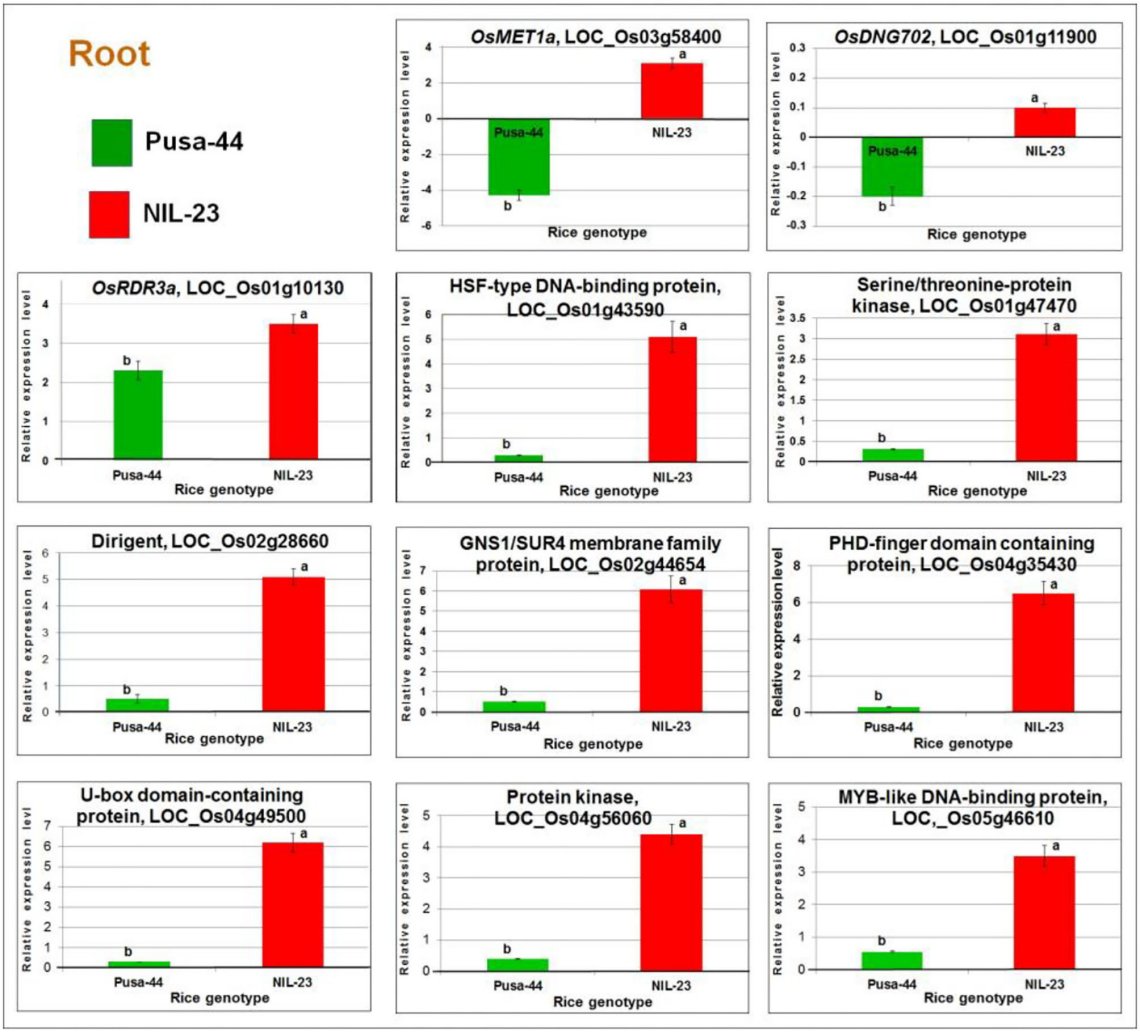
RT-qPCR validation of randomly selected differentially expressed or methylated genes. cDNA was prepared for the total RNAs isolated from roots of 45-day-old plants of the contrasting rice genotypes Pusa-44 (P-deficiency stress-sensitive) and NIL-23 (stress-tolerant) grown hydroponically under control (16 ppm inorganic phosphorus) and P-starvation (0 ppm P) stress. Mean values (*n* = 9) followed by different lowercase letters are significantly different (*p* ≤ 0.05). The error bars represent standard deviation (±SD).

The RT-qPCR analysis of *OsMET1a* [DNA methyltransferase 1 (MET1) responsible for CG methylation] and *DNG702* (DNA glycosylase for DNA demethylation) confirmed their upregulated expression due to hypomethylation of the promoter in root of NIL-23 but a downregulated expression of the gene due to hypermethylation in root of Pusa-44 under the stress. Similarly, RT-qPCR analysis of *OsRDR3a* (RNA-dependent RNA polymerase for RNA-directed DNA methylation) confirmed its significantly higher upregulated expression due to hypomethylation of the promoter in root of NIL-23 under the stress but lesser upregulated expression in root of Pusa-44 following the effect of hypomethylation. The expression level of *OsDNMT2* (for CHH-specific methylation), *DNG701* (a DNA glycosylase), and *OsRDR6* (an RNA-dependent RNA polymerase responsible for RNA-directed DNA methylation) was also validated, which indicated to follow the downregulated pattern of the genes (as detected in the RNA-seq analysis) due to hypermethylation of the promoter. Similarly, the RT-qPCR validation of eight randomly selected DEGs (affected by P-starvation stress-induced DNA demethylation) confirmed the consistency in expression pattern (upregulation) of the genes observed in the RNA-seq analysis (Figure 12).

## Discussion

Several adaptive mechanisms are taken up by plants to cope with P-starvation stress, which includes transcriptional reprograming to alter morphological, physiological, and biochemical responses to improve the uptake, mobilization, and reutilization of P (Kumar et al., 2021b, 2022). Our comparative methylome and transcriptome analyses of a high-yielding rice (Pusa-44) cultivar and its near-isogenic line (NIL-23, Pusa-44 introgressed with *Pup1*) grown under P-starvation stress indicate that *Pup1* QTL play the important role in regulating the expression of phosphate-starvation responsive and RSA-related genes through modulating DNA methylation in different parts of the gene under the stress. With the limited knowledge available so far about the genes on *Pup1* QTL and their functions, we could decipher the roles of the QTL in regulating gene expression by altering chromatin architecture or epigenetic landscape.

The requirement of P increases with the advancing growth or biomass of plant, and the plant adopts several other mechanisms, in addition to modulation in RSA, to maintain P homeostasis. Therefore, we used 45-day-old (vegetative/tillering stage) plants (Kumar et al., 2021b, 2022) to understand the role of DNA (de)methylation in modulating the expression of P-starvation responsive genes. The changes in DNA methylation landscape due to the introgression of *Pup1* QTL and induced by P-starvation stress were assessed in root and shoot of the contrasting rice (Pusa-44 and NIL-23) genotypes with a sufficiently high-coverage (~25 ×) and >50% mapping efficiency of the bisulfite sequencing data (Supplementary Table S2), which indicated trustworthiness of the WGBS data. Higher 5-mC content in root (compared to that in shoot), which decreased under the stress, indicates the existence of 5-mC to the extent of ~4.5%; hence, it can be rightly considered as the 5th base in the genome. About 15% of the total cytosines were present in their epigenetically modified (5-mC) form (Figure 1). A significant increase in methylation in all the three (CG, CHG, and CHH) contexts in shoot of NIL-23 (the rice genotype harboring *Pup1* QTL) and decrease in methylation in CG and CHG contexts (but increase in CHH context) in shoot of Pusa-44 were observed (Figure 2A). A significant decrease in methylation in CG and CHG contexts but only marginal decrease in CHH context in root of Pusa-44, while a marginal (0.2–0.6%) increase in methylation in all the three (CG, CHG, and CHH) contexts in root of NIL-23 was observed (Figure 2B), in response to the P-starvation stress, demonstrates the role of the *Pup1* QTL in epigenetic modification in modulating gene expression under the stress. Our earlier reports (Kumar et al., 2021b, 2022) clearly indicated differential expression of genes in root and shoot of plant playing important roles in P uptake and P assimilation, respectively. These encouraged us to decipher the role of DNA (de)methylation, as one of the epigenetic machinery, in regulation of gene expression under P-starvation stress.

Maximum (75%) of the DmCs were mapped in the promoter, whereas some of them were also mapped in the gene body (4–8%), downstream region (3–4%), and distal intergenic region (11–13%) in root of NIL-23 under the stress (Figure 3), which confirmed the important role of promoter in epigenetic regulation of gene expression. This is in agreement with the earlier reports (Borgel et al., 2010; Bhatia et al., 2018; Kang et al., 2019; Grzybkowska et al., 2020; Kumar and Mohapatra, 2021). A significant change in DNA methylation in the promoter region, particularly in the proximal (<1 kb) promoter, due to P-starvation stress confirmed the importance/role of epigenetic modification in regulating gene expression under environmental stress. Such observations are in agreement with that reported by several earlier authors (Hashimoto et al., 2013; Mager and Ludewig, 2018; Grzybkowska et al., 2020). A higher number (16,536) of hypomethylated DMRs in root of NIL-23 (compared to 13,505 hypomethylated DMRs in root of Pusa-44 and 14,322 hypermethylated DMRs in root of NIL-23), as well as the higher number of DMRs in all the three [CG (6,122), CHG (4,346), and CHH (6,068)] sequence contexts in root of NIL-23 under the stress (Figure 4B), indicate the association between hypomethylation of DNA and upregulated expression of the stress-responsive genes, which has also been reported earlier in plants (Wada et al., 2004; Rauluseviciute et al., 2020; Wang et al., 2021a). The occurrence of a higher number of DMRs in CHH context (compared to that in other contexts) in shoot and root of the rice genotypes is in agreement with that reported by Mager and Ludewig (2018) in maize under nutrient deficiency stress.

The lesser number (4,820) (26.5%) of hypermethylated DMRs in root of NIL-23, compared to 5,009 (31.5%) hypermethylated DMRs in CHH context in root of Pusa-44, indicates the role/importance of hypermethylation in regulation of gene expression and providing stress tolerance (Figure 5A). Significantly, a higher number of hypomethylated (5,527) DMRs in CHH context in root of NIL-23, compared to 4,950 hypomethylated DMRs in root of Pusa-44, indicate the importance of hypomethylation in root of NIL-23 in making it stress-tolerant. Similarly, the higher number (5,527, 33.8%) of hypomethylated DMRs in CHH context in root, compared to 4,503 (27.5%) hypomethylated DMRs in shoot of NIL-23, indicates the role of hypomethylation or upregulated expression of the genes in root in providing stress tolerance. A higher number of hypomethylated (5,527) DMRs in CHH context in root of NIL-23, compared to 4,950 hypomethylated DMRs in root of Pusa-44, confirm the importance of hypomethylation in root of NIL-23 under P-starvation stress in providing stress tolerance (Figure 5B). While most of genes possessed only one DMR, some of the genes contained multiple DMRs. Such findings corroborate with those of Secco et al. (2015), which reinforce the association between differential DNA methylation and gene expression under P-starvation.

The comparative analysis of changes in DNA methylation level in response to P-starvation stress provided insight into the plasticity of genome to be epigenetically shaped by nutrient deficiency. Although our approach of analyzing the stress-induced changes in DNA methylation in response to P-starvation stress provides a snapshot of the methylation landscape in different tissues, our experimental design enabled us to decipher the molecular functions of the *Pup1* QTL in regulating gene expression under the stress. Moreover, our analysis of the stress-induced changes in DNA methylation landscape indicated a different epigenetic status and gene expression level in different tissues under the stress due to the *Pup1* QTL. About 20% change in DMRs in NIL-23 under the stress was observed due to the introgression of *Pup1* QTL in Pusa-44 genetic background, and such a study has not yet been reported in plant.

The regulatory roles of DNA methylation on gene expression have been widely studied, and methylation of promoter has generally been associated with repression of gene expression, whereas gene body methylation may enhance or repress the gene expression (Zilberman et al., 2007; Li et al., 2012; Wang et al., 2013). Our study on comparative methylome and transcriptome analyses of two contrasting rice genotypes revealed interesting relationships between DNA methylation and gene expression level, which appears to be more complex than it is expected. Our observation on 328 DEGs to be upregulated due to hypomethylation in CHH context, whereas 102 DEGs to be downregulated due to hypermethylation in root of NIL-23, compared to 178 DEGs upregulated due to hypomethylation and 199 DEGs downregulated due to hypermethylation in root of Pusa-44 under the stress, indicates that only a small number of genes were regulated through epigenetic (DNA methylation) modification under P-starvation stress. In view of the current information about the genes located on the *Pup1* QTL, neither the genes associated with P uptake (Heuer et al., 2009) nor the genes for epigenetic modifications have been identified on the QTL. However, out of the 68 gene models predicted on the *Pup1* QTL, many of them show sequence similarity with transposons. Some of the genes code for putative fatty acid oxygenase, dirigent-like protein, aspartic proteinase, hypothetical proteins, and putative protein kinase. These findings on the difference in DNA methylation not showing a correlation with the gene expression level agree with the findings of several earlier studies (Garg et al., 2015; Secco et al., 2015; Wang et al., 2015; Mager and Ludewig, 2018). Variation in DNA methylation affects gene expression, directly or indirectly and in a coordinated manner through different other epigenetic modulators such as histone modifications and chromatin architecture (Wada et al., 2004; Park et al., 2018; Kumar et al., 2021a; Wang et al., 2021a).

We observed negative as well as positive correlations between DNA methylation and gene expression levels. A negative correlation between methylation and gene expression levels was observed for 2.5% of the DMR-associated genes in root of NIL-23, which corroborates with the findings of Zilberman et al. (2007), Li et al. (2012), Vining et al. (2015), and Anastasiadi et al. (2018). However, a positive correlation between methylation (in CG, CHG, and CHH contexts) and gene expression levels was also observed for >2% of the DMR-associated genes in root of NIL-23, which corroborate with the findings of Gent et al. (2013), Secco et al. (2015), Song et al. (2015), and Wang et al. (2021a). A positive correlation between CHH hypermethylation and expression of genes has been reported earlier in maize (Gent et al., 2013), rice (Secco et al., 2015), cotton (Song et al., 2015), and soybean (Chu et al., 2020). DNA methylation in an unsymmetric CHH context is generated or established *de novo* with the help of small-interfering (RdDM) pathway. Our finding on the upregulated expression of RNA-dependent RNA polymerase (LOC_Os01g10130, *OsRDR3a*) under the stress in the rice genotypes (Supplementary Table S19) corroborates with the P-starvation-induced hypermethylation of promoters or genes in CHH context mediated by RdDM pathway in rice (Secco et al., 2015) and soybean (Chu et al., 2020).

Differentially methylated regions in different genic regions have been reported to have different effects on gene expression (Liang et al., 2011; Dubin et al., 2015), indicating no direct or linear relation between methylome and transcriptome. Moreover, the effects of DNA methylation on gene expression are tissue- or organ-specific (Gutierrez-Arcelus et al., 2015) and depend on the developmental stage of plant as well as the environmental conditions (Wang et al., 2015; Yong-Villalobos et al., 2015). Interestingly, the changes in DNA methylation under P-starvation stress in NIL-23 occurred primarily in the genes coding for the proteins associated directly or indirectly with stress tolerance, which is in agreement with the findings of earlier studies (Zilberman et al., 2007; Wang et al., 2014b; Colicchio et al., 2015).

Further analysis of differential methylation in the CHH context and its effect on gene expression indicated its role in modulating several GO terms (biological/cellular/molecular processes) under the stress. A considerably higher (34) number of GO terms (e.g., primary metabolic process, macromolecule metabolic process, nitrogenous compound metabolic process, and nucleic acid-binding proteins, etc.) were over-represented in root of NIL-23 due to hypomethylation of genes in CHH context (Figures 6A, 7A) whereas hypermethylation of genes for 27 GO terms (e.g., regulation of macromolecule metabolic process, regulation of the cellular metabolic process, regulation of nitrogenous compound metabolic process, regulation of gene expression, and regulation of transcription) in root of Pusa-44 (Figures 6B, 7B) indicates the adaptive roles of *Pup1* QTL in making NIL-23 tolerant to P-starvation stress.

Many P-responsive genes, some of the genes for P transporters, TFs, and genes for DNA methylation and histone modification were regulated through modulation in DNA methylation in root of NIL-23 (Supplementary Table S3), indicating epigenetic modulation of the stress-responsive gene expression in P uptake mediated by the *Pup1* QTL. In plants, laccases have been reported to be associated with the biosynthesis and polymerization of lignin (Zhao et al., 2013), elongation (Balasubramanian et al., 2016), and response to different stresses (Liu et al., 2017). Our observation on 2.5-fold upregulated expression of laccase (LOC_Os01g62600) in root of NIL-23 due to hypomethylation of the gene (but hypermethylation and downregulation of the gene in root of Pusa-44) indicates its role in P-starvation stress tolerance (Supplementary Table S3). Multicopper oxidase (MCO) has been reported to be pivotal for eliciting inhibition of primary root growth, and thus promoting lateral roots, during P-deficiency stress in Arabidopsis (Svistoonoff et al., 2007). Our observations on the upregulated expression of several genes for MCOs due to hypomethylation of the genes in CG, CHG, and CHH contexts in roots of NIL-23 indicate their role in P-starvation stress tolerance (Supplementary Tables S5, S10, S15). A gene for BAG domain-containing protein (LOC_Os09g35630) showing upregulated expression in root of NIL-23 due to hypomethylation in CHH context in promoter reiterates the role of *Pup1* QTL in providing P-starvation stress tolerance to NIL-23 (Supplementary Table S5).

Moreover, the P-responsive genes such as serine–threonine protein kinase, phosphoesterase, phosphoglycerate kinase, glycerol-3-phosphate dehydrogenase, dirigent, expansin, phosphate-induced protein 1, purple acid phosphatase, monogalactosyldiacylglycerol synthase 2, and amino acid transporters, and so on. were hypomethylated and upregulated in roots of NIL-23 under P-starvation stress (Supplementary Table S5). However, a different set of genes, including Shikimate kinase, sucrose-phosphate synthase, cyclin-dependent kinase, phosphoribosyl transferase, phosphatidylinositol-4-phosphate 5-kinase, phosphatidylinositol kinase, ATPase, fructose-1,6-bisphosphatase, glyceraldehyde-3-phosphate dehydrogenase, hexokinase, ATP-dependent RNA helicase, DNA-directed RNA polymerase I, phosphoribosyl transferase, photosystem I reaction center, aminotransferases, etc., hypermethylated in root of NIL-23 were downregulated, indicating the role of epigenetic modification on gene regulation (Supplementary Table S6). Moreover, hypomethylation in CHH context and upregulated expression of 489 genes in shoot of NIL-23, whereas hypermethylation and downregulation of 140 genes in shoot of Pusa-44 (Figure 8A) indicated their roles in maintaining P homeostasis. Several genes epigenetically regulated through (de)methylation in the proximal promoter region (Supplementary Table S4), hypomethylated in promoter region causing upregulated expression, and hypermethylation of gene body in CHH context (Supplementary Table S17) or CG context (Supplementary Table S18) caused up-/downregulated expression of genes.

Hypomethylation of promoter in CHH context in root caused upregulated expression of the gene (Figure 9A, Supplementary Figure S2A), and hypomethylation of both promoter and gene body caused upregulated expression of genes in NIL-23 (Figure 9C, Supplementary Figure S2C) but downregulated expression of LOC_Os01g71280 in root of Pusa-44 (Figure 9D), whereas hypermethylation of both promoter and gene body caused downregulated expression of (LOC_Os01g47470) the genes in NIL-23 (Supplementary Figure S2D), indicating differential interaction of methylation levels of promoter and the gene body. Hypomethylation of gene body in CHH context in root caused upregulated expression of a gene (LOC_Os04g03050) in NIL-23 under P-starvation stress (Figure 9E) but downregulated expression of the gene in root of Pusa-44 (Figure 9F). Similarly, hypermethylation of gene body of LOC_Os02g19924 under control conditions caused upregulated expression in root of NIL-23 (Supplementary Figure S2E), but hypomethylation of the gene under P-starvation stress caused no significant difference in its expression level in root of Pusa-44 (Supplementary Figure S2F), indicating the involvement in other regulatory mechanisms in controlling gene expression under the stress. All of these point out complex regulatory principles of epigenetic [cytosine (de)methylation as well as other components] modifications depending on the location/combination. These findings are in agreement with those of Chan et al. (2005) and Li et al. (2012). H2A.Z, one of the H2A variants, has important implications in regulation of gene expression, DNA repair, genome stability, recombination, cell cycle, female meiosis, and response to different abiotic and biotic stresses. H2A.Z is typically enriched at the first nucleosome at transcriptional start site (+1 nucleosome), whereas it is found at a lower level in the gene body (Zilberman et al., 2008). It has been proposed that the existence of H2A.Z in +1 nucleosome is associated with activation of transcriptional activity, whereas it has a repressive effect on transcriptional activity when present in the gene body (Sura et al., 2017). Swi/Snf2-related 1 (SWR1, a chromatin remodeler) complex has been reported to help regulating transcription of a number of PSR genes through deposition of H2A.Z histone variant at the target genes (Lindstrom et al., 2006). Smith et al. (2010) reported the role of SWR1 complex in deposition of H2A.Z in modulating the expression of PSR genes in Arabidopsis. We observed differential expression of *SWR1* genes in the rice genotypes under the stress (data not shown) which emphasize the complexity of epigenetic regulatory mechanisms.

Upregulated expression of transcription factors such as HSF-type DNA-binding domain-containing protein, MYB family transcription factors, no apical meristem protein, homeobox-associated leucine zipper, helix–loop–helix DNA-binding domain-containing protein, OsMADS, and so on. due to hypomethylation of the promoter and downregulated expression of ethylene-responsive transcription factor 2 due to hypermethylation of promoter in root of NIL-23 under the stress are in agreement with those of Zheng et al. (2009), Thibaud et al. (2010), and Nagarajan and Smith (2012) (Supplementary Table S9). Hypomethylation or upregulated expression of several important genes in CHG context, including MYB family transcription factor (LOC_Os01g12860), AMP-binding enzyme, AP2 domain-containing protein, MCO domain-containing proteins (LOC_Os02g50150, LOC_Os03g52475, and LOC_Os05g38030), serine carboxypeptidase, PHD-finger domain-containing protein, gibberellin 20 oxidase 2, SPX domain-containing protein, cyclin-dependent kinase inhibitor, etc. (Supplementary Table S10), in root of NIL-23 might be responsible for providing P-starvation stress tolerance to this genotype due to the introgression of *Pup1* QTL. The up- or downregulated expression of genes for MCOs, amino acid permease family proteins, protein kinases, etc. due to hypo-/hypermethylation of DNA in CG context in promoter or gene body, observed in root of NIL-23 under the stress (Supplementary Tables S11, S12), agrees with the earlier reports (Wada et al., 2004; Mager and Ludewig, 2018; Wang et al., 2021a). Moreover, a positive effect of methylation of promoter on the expression of the gene has also been reported in plants (Williams et al., 2015; Moreno-Romero et al., 2016). Effect of the location of DMR in some of the genes (e.g., MYB transcription factor, serine– threonine protein kinase receptor, PDH-finger domain-containing protein, annexin, tubulin/FtsZ domain-containing protein, vacuolar protein sorting–protein 4B, DNA-binding protein, stress-induced protein, and BAG domain-containing protein, etc.) on their expression level was observed in roots of Pusa-44 and NIL-23 (Supplementary Tables S14, S15). Some of the so far unannotated, expressed genes observed to be hypermethylated or downregulated in root of NIL-23 (but hypomethylated or upregulated in root of Pusa-44) (Supplementary Table S16) might be the coding for the novel regulatory proteins playing important role(s) in tolerance to P-starvation stress.

The DNA methyltransferases and DNA demethylases play a crucial role in maintaining DNA methylation level during plant development and environmental stress. Methylation of cytosine or demethylation of 5-mC is altered by changes in the expression level of DNA methyltransferase or DNA demethylase genes. Our observation on the expression level of RdDM pathway and DNA (de)methylation-related genes in root of NIL-23 under P-starvation stress confirmed the roles of epigenetic mechanism in the regulation of the stress-responsive genes, due to the introgressed *Pup1* QTL, in providing P-starvation stress tolerance (Supplementary Table S19). Our findings on the upregulated expression of *MET1a, CMT1, CMT3, DRM1* (involved in DNA methylation), *DNG702* (involved in DNA demethylation), and *RDR3* (involved in RNA-directed DNA methylation) and downregulated expression of *MET1b* (responsible for maintaining CG methylation), *CMT2* (responsible for maintaining CHG methylation), *DRM2, DRM3, DNMT2* (involved in CHH-specific methylation), *DML3, DNG701, DNG704* (involved in DNA demethylation), *RDR2*, and *RDR6* (involved in RNA-directed DNA methylation) corroborate with the findings of Moritoh et al. (2012), Wang et al. (2014c), Yong-Villalobos et al. (2015), Feng et al. (2016), Lu et al. (2016), and Li et al. (2020). Interestingly, the methylation level of the genes coding for DNA (de)methylases and RDR polymerases varied significantly in their promoter regions, affecting their expression (Supplementary Table S20). Methylation of gene body caused up-/downregulated expression of the gene, which corroborates with the findings of Wang et al. (2014a); however, a combination of promoter and gene body methylation caused a mild repressive effect on gene expression (Supplementary Table S20). Thus, we propose that the *Pup1* QTL might harbor the genes (yet to be annotated/identified) responsible for sensing the stress, modulating expression of the enzymes involved in epigenetic alterations to regulate gene expression under P-starvation or deficiency stress.

**Figure 13:**
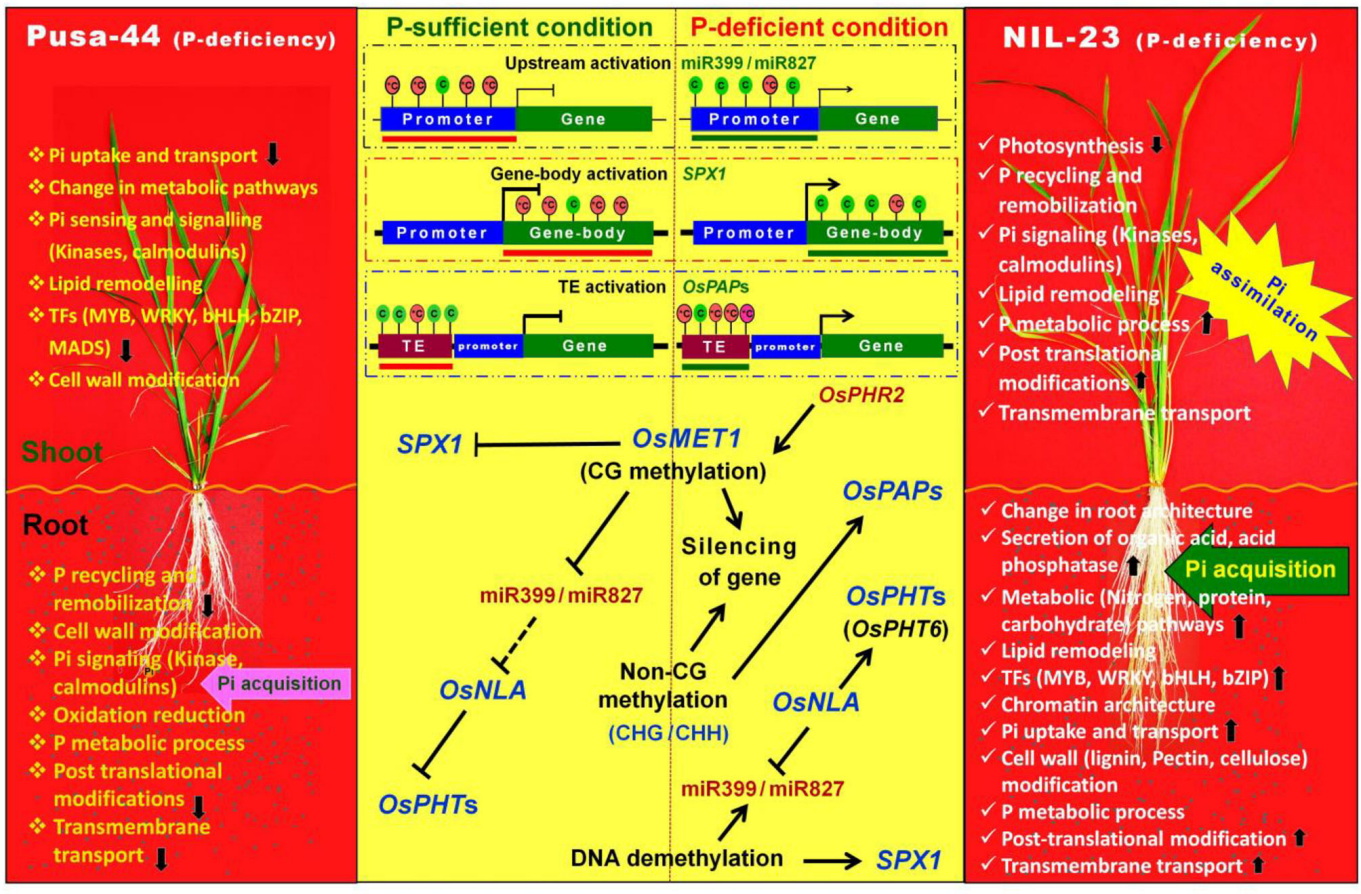
Diagrammatic representation of epigenetic regulation of gene expression through DNA (de)methylation in rice (Pusa-44 stress-sensitive, and NIL-23 stress-tolerant due to introgression of *Pup1* QTL) genotypes under P-starvation stress. Root plays an important role in acquisition of Pi, whereas shoot helps assimilation of Pi. The upward arrow (↑) indicates upregulated expression, whereas the downward arrow (↓) indicates downregulated expression of the associated genes. Methylation status at upstream region, gene body, and TE affects gene expression. Red lollipops depict hypermethylated (5-methylcytosine (*C) positions, whereas green lollipops represent hypomethylated (C= cytosine) positions. Red line indicates inactive transcriptional states, whereas green line denotes active transcriptional state. Under P-deficiency condition, a set of P-starvation–responsive genes is activated *via* key regulator PHR2. SPX, SYG1/Pho81/XPR1 domain proteins; MET, methyltransferase; PHT; phosphate transporter; PAP, purple acid phosphatase; NLA, nitrogen limitation adaptation.

Thus, this study is the first comprehensive analysis of the molecular functions of *Pup1* QTL, particularly its role in epigenetic regulation of gene expression, in providing tolerance to P-starvation stress in rice (NIL-23). Based on our findings, we depict a model wherein DNA methylation impacts the expression of a set of PSR genes during P-sufficient and P-deficient conditions (Figure 13). MET1 maintains methylation or silencing states of PSR genes (e.g., miR399/miR827). Under P-deficient conditions, PHR2 activates PSR genes, including *MET1* (Yong-Villalobos et al., 2015). PHR2 might also modulate the expression of other DNA methyltransferase genes such as *DRM, CMT2, CMT3*, and demethylase (*ROS1*), which modulate the expression of PSR genes. Such dynamic methylation is tangled with P transport, lipid remodeling, and P recycling processes, which are important for P homeostasis and mediated through the introgression of *Pup1* QTL in NIL-23. Further investigations on the candidate gene(s) for DNA methylation-regulated P-starvation responses, hypo-/hypermethylated DMRs (epimarks), and epigenome editing of plants (Kumar, 2019) would be of great interest for epigenetic manipulation of crop plants in future. Moreover, the impacts of other epigenetic factors such as histone modifications, non-coding RNAs, chromatin architecture (Hu et al., 2021; Jabre et al., 2021), and their interactions with DNA methylation marks (Kumar and Mohapatra, 2021) need to be deciphered before epigenetic regulatory mechanism(s) can be successfully deployed in crop improvement programs.

## Data Availability Statement

The datasets presented in this study can be found in online repositories. The names of the repository/repositories and accession number(s) can be found below: NCBI Sequence Read Archive (SRA) database under the BioProject ID PRJNA802863, NCBI Sequence Read Archive database under the BioProject ID PRJNA667189.

## Author Contributions

SuK and TM conceived the experiments. KV developed the near-isogenic lines, evaluated them, and provided the best performer for experimentation. KS carried out the experiments. SaK performed bioinformatic analyses. SuK, KS, and SaK prepared the initial draft. TM and VC provided the inputs for the revision of the manuscript. SuK and SaK revised the manuscript. SuK, VC, and TM finalized the manuscript. All authors approved the final draft for publication.

## Funding

The research was carried out with financial support from Extramural Research grants [18(3)/2018-O&P] from the Indian Council of Agricultural Research, Government of India, New Delhi.

## Conflict of Interest

SaK is employed by Decode Genomics Private Limited, New Delhi, India. The remaining authors declare that the research was conducted in the absence of any commercial or financial relationships that could be construed as a potential conflict of interest.

## Publisher's Note

All claims expressed in this article are solely those of the authors and do not necessarily represent those of their affiliated organizations, or those of the publisher, the editors and the reviewers. Any product that may be evaluated in this article, or claim that may be made by its manufacturer, is not guaranteed or endorsed by the publisher.
