# Supplementary material for: Pup1 QTL Regulates Gene Expression Through Epigenetic Modification of DNA Under Phosphate Starvation Stress in Rice
Source: Front Plant Sci. 2022 May 31;13:871890. doi: 10.3389/fpls.2022.871890 (PMC9195100; doi:10.3389/fpls.2022.871890)

Supplementary Figure S1 | Representative genome browser screenshots showing effects of DNA methylation on gene expression by hyper-hypo-methylation in CHH context in contrasting rice (NIL-23 and Pusa-44) genotypes grown hydroponically at 16 ppm or 0 ppm Pi. (A) hypomethylation of promoter caused up-regulated expression gene encoding protein-kinase domain-containing protein in root of NIL-23 under the stress, (B) no significant change in methylation of the promoter, but down-regulated expression of the gene was observed in root of Pusa-44. (C) hypomethylation of promoter, as well as the gene-body of serine/threonine-protein kinase gene caused up-regulated expression in root of NIL-23 under the stress, (D) hypermethylation of promoter as well as the gene-body caused down-regulated expression of the gene in root of Pusa-44 under the stress. (E) hypermethylation of gene-body of aminotransferase gene caused up-regulated expression of the gene in root of NIL-23 under control condition, (F) hypomethylation of the gene under stress did not result in any change in its expression in root of Pusa-44. The blue bars (in upper 2 panels) represent methylation level (DMRs), and the expression level of gene is represented with red bars. Ctrl= control, Trt= P-starvation stress treatment.

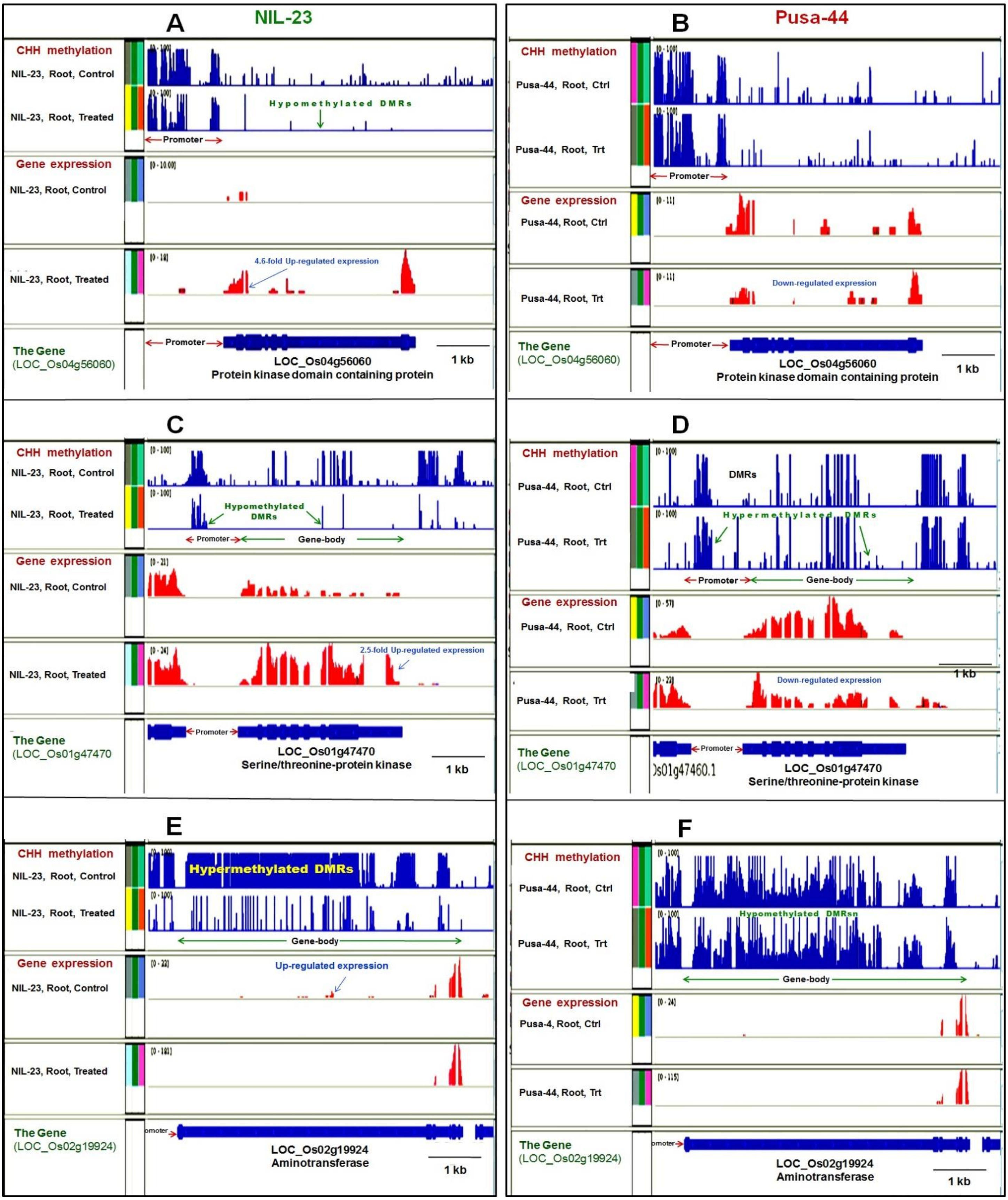

Supplementary Figure S2 | GO enrichment analysis of gene clusters showing either hypermethylation (2-fold, P<0.05) in CHG context in promoter region and down-regulated (2-fold, P<0.05) expression or hypomethylation (2-fold, P<0.05) and up-regulated expression (2-fold, P<0.05) in root and shoot of rice (NIL-23, P-deficiency tolerant and Pusa-44, P-deficiency sensitive) genotypes. Only top 20 clusters of genes have been depicted here. FDR was calculated based on P-value from the hypergeometric test. Fold enrichment was defined as percentage of genes in the list belonging to a pathway, divided by the corresponding percentage in the background.

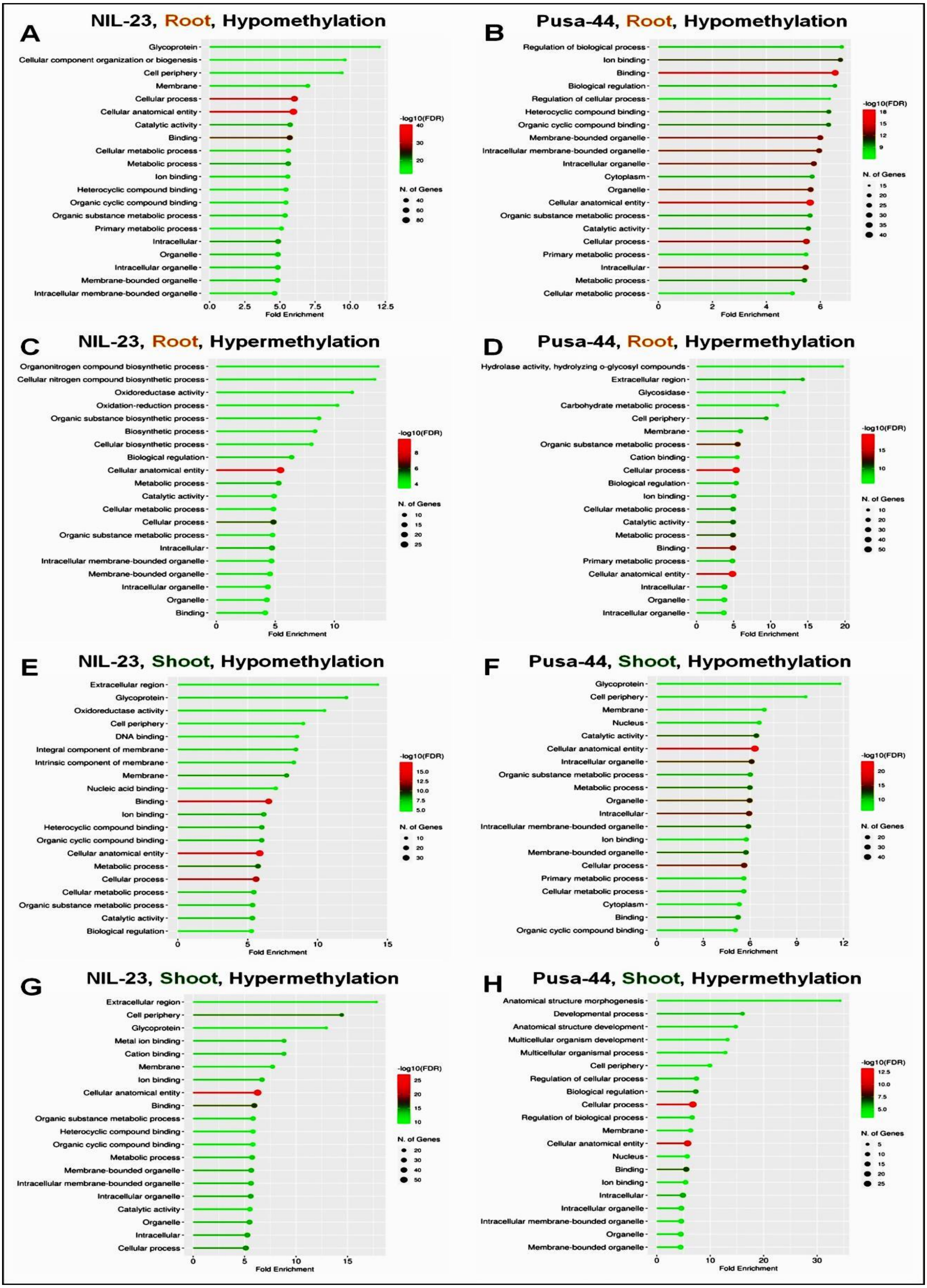

Supplement: Supplementary file 1 [file Data_Sheet_1.zip › Figure S1 and S2.PDF]
