# Supplementary material for: Pup1 QTL Regulates Gene Expression Through Epigenetic Modification of DNA Under Phosphate Starvation Stress in Rice
Source: Front Plant Sci. 2022 May 31;13:871890. doi: 10.3389/fpls.2022.871890 (PMC9195100; doi:10.3389/fpls.2022.871890)
Supplement: Supplementary file 1 [file Data_Sheet_1.zip › Table S1 and S2.doc]

**Supplementary** **Table S1.** The list of primers used for RT-qPCR validation of some of the randomly selected differentially expressed genes in the contrasting rice (Pusa-44, P-deficiency stress sensitive; NIL-23, P-deficiency tolerant) genotypes.

| **Gene Name** | **Gene ID** | **Forward Primer (5′→ 3′)** | **Reverse Primer (5′→ 3′)** |
| --- | --- | --- | --- |
| *OsMET1a* | LOC_Os03g58400 | TCTGTAACGGATCCTCAGCC | TGCTTGTTCTGGATGTTGCC |
| *OsDNG702* | LOC_Os01g11900 | GTGGTTGCGCTATGTCGATT | GACGCACACACTCAACACTT |
| *OsRDR3a* | LOC_Os01g10130 | CAAACCTTCTGAACCGTGGG | TATCAGCTGCCGTACCAAGT |
| HSF-type DNA-binding protein | LOC_Os01g43590 | CCCAGTACACCAGCTTCACC | TCTTCTTCCATCTGCAAGCTC |
| Serine/threonine-protein kinase | LOC_Os01g47470 | GGGATGGGGTCACTGGTTTA | CTTCACGGCAACTTCAGTCC |
| Dirigent | LOC_Os02g28660 | CCGCCCATTCTAAGTCATGC | CCTCTTGTGTGACTGGGAGT |
| GNS1/SUR4 membrane family protein | LOC_Os02g44654 | GACTCGTACAAGTTCGTCGC | GAGGGACATCTTCTGCTCGA |
| PHD-finger domain containing protein | LOC_Os04g35430 | TTTCTTGTGCAGTGGACAGC | AGTTTTGCCAAGACGACGAC |
| U-box domain-containing protein | LOC_Os04g49500 | GATGAACGGAGCAGACAACC | TCTGTGTCGCTCTGGAGATC |
| Protein kinase | LOC_Os04g56060 | CCGAGACAATATGGTGGGGA | GAGCTCCGTCAAGAAGTCCT |
| MYB-like DNA-binding protein | LOC,_Os05g46610 | TCCCGAGGAAGACGATAAGC | GGCCTGAGGTAGTTGATCCA |
| *Actin* gene | LOC_Os03g50885 | TTGCTGACAGGATGAGCAAG | TGGAATGTGCTGAGAGATGC |
| *β-tubulin* gene | LOC_Os01g59150 | GCTGACCACACCTAGCTTTGG | AGGGAACCTTAGGCAGCATGT |

**Supplemental Table S2:** Summary of methylome (WGBS) data mapping statistics

| **Sample ID** | **Description^*^** | **Total read pairs** | **Trimmed reads** | **Unique reads** | **Mapping efficiency** |
| --- | --- | --- | --- | --- | --- |
| PSF | Pusa-44, Shoot, Full (16 ppm) Pi | 35038239 | 33421516 | 19195157 | 57.4% |
| PSZ | Pusa-44, Shoot, Zero (0 ppm) Pi | 33456032 | 31667582 | 17950989 | 56.7% |
| PRF | Pusa-44, Root, Full (16 ppm) Pi | 47224097 | 44186139 | 23153536 | 52.4% |
| PRZ | Pusa-44, Root, Zero (0 ppm) Pi | 45746874 | 42376080 | 21527048 | 50.8% |
| NSF | NIL-23, Shoot, Full (16 ppm) Pi | 29221246 | 28026977 | 16010306 | 57.1% |
| NSZ | NIL-23, Shoot, Zero (0 ppm) Pi | 39662388 | 38322894 | 22211571 | 58.0% |
| NRF | NIL-23, Root, Full (16 ppm) Pi | 45183171 | 43620336 | 21947029 | 50.3% |
| NRZ | NIL-23, Root, Zero (0 ppm) Pi | 38055007 | 36473703 | 18233325 | 50.1% |

^*^The rice plants were grown hydroponically in PusaRicH medium containing full (16 ppm) inorganic phosphorus (Pi) or no/zero (0 ppm) Pi in the medium.
